# Differential subgenome expression underlies biomass accumulation in allotetraploid *Pennisetum giganteum*

**DOI:** 10.1186/s12915-023-01643-w

**Published:** 2023-07-21

**Authors:** Longsheng Xing, Meijia Wang, Qiang He, Hongyu Zhang, Hanfei Liang, Qinghong Zhou, Yu Liu, Ze Liu, Yu Wang, Cailian Du, Yao Xiao, Jianan Liu, Wei Li, Guixia Liu, Huilong Du

**Affiliations:** 1 College of Life Sciences, Institute of Life Sciences and Green Development, Hebei University, Baoding, 071000 China; 2 Hebei Basic Science Center for Biotic Interaction, Baoding, 071000 China

**Keywords:** *Pennisetum giganteum*, Genome evolution, Polyploid advantage, Subgenome dominance, C4 photosynthesis

## Abstract

**Background:**

*Pennisetum giganteum* (AABB, 2n = 4x = 28) is a C4 plant in the genus *Pennisetum* with origin in Africa but currently also grown in Asia and America. It is a crucial forage and potential energy grass with significant advantages in yield, stress resistance, and environmental adaptation. However, the mechanisms underlying these advantageous traits remain largely unexplored. Here, we present a high-quality genome assembly of the allotetraploid *P. giganteum* aiming at providing insights into biomass accumulation.

**Results:**

Our assembly has a genome size 2.03 Gb and contig N50 of 88.47 Mb that was further divided into A and B subgenomes. Genome evolution analysis revealed the evolutionary relationships across the Panicoideae subfamily lineages and identified numerous genome rearrangements that had occurred in *P. giganteum*. Comparative genomic analysis showed functional differentiation between the subgenomes. Transcriptome analysis found no subgenome dominance at the overall gene expression level; however, differentially expressed homoeologous genes and homoeolog-specific expressed genes between the two subgenomes were identified, suggesting that complementary effects between the A and B subgenomes contributed to biomass accumulation of *P. giganteum.* Besides, C4 photosynthesis-related genes were significantly expanded in *P. giganteum* and their sequences and expression patterns were highly conserved between the two subgenomes, implying that both subgenomes contributed greatly and almost equally to the highly efficient C4 photosynthesis in *P. giganteum*. We also identified key candidate genes in the C4 photosynthesis pathway that showed sustained high expression across all developmental stages of *P. giganteum*.

**Conclusions:**

Our study provides important genomic resources for elucidating the genetic basis of advantageous traits in polyploid species, and facilitates further functional genomics research and genetic improvement of *P. giganteum*.

**Supplementary Information:**

The online version contains supplementary material available at 10.1186/s12915-023-01643-w.

## Background

Grasses provide most of the calories in human diets and are the most abundant but still not fully exploited sustainable resource [1–3]. *Pennisetum* species are widely distributed worldwide and many of them are C4 plants, which generally exhibit rapid growth, high arid tolerance, and high biomass production [4, 5]. *Pennisetum giganteum* is native to Africa and is well known for its huge biomass and production [6, 7]. *P. giganteum* can grow in tropical, subtropical, and temperate regions and has been cultivated in more than 30 provinces in China and up to 80 countries worldwide [8]. *P. giganteum* is a typical C4 plant with a solar energy conversion rate that is 4–7.5 times that of broad-leaved trees; they are generally grown to a height of 3–5 m and the maximum height can be up to 7.08 m [9]. The annual yield of fresh *P. giganteum* grass was reported to be up to 200–400 tons per hectare, with crude protein content of 10.8% after 4 weeks of growth. The cellulose-rich *P. giganteum* can be used for biomass power generation and for producing nano-cellulose, which is a raw material of non-wood fiber that is suitable for pulping and papermaking [7, 10]. Therefore, *P. giganteum* is regarded as a high-yield and high-quality forage grass and an extremely important raw material for bioenergy generation. The synthesis of cellulose is mainly mediated by cellulose synthase (*CesA*) complexes (CSCs) that is located on the plasma membrane [11, 12]. In monocots, the *CesA* gene superfamily are mainly composed of *CesA* and seven cellulose synthase-like (*Csl*) clades, such as *CslA*, *CslC*, *CslD*, *CslE*, *CslF*, *CslH*, and *CslJ* [12–14]. It was widely accepted that the *CesA* genes are mainly participating in cellulose synthesis and the *Csl* genes mainly encode enzymes that synthesize hemicellulose [14, 15]. Due to their essential roles in cell wall synthesis, the members of this superfamily have been extensively investigated since the discovery [11, 13, 14, 16–18]. However, the systematic identification and expression profiling of *CesA*/*Csl* genes in *P. giganteum* are still unexplored.

**Fig. 1:**
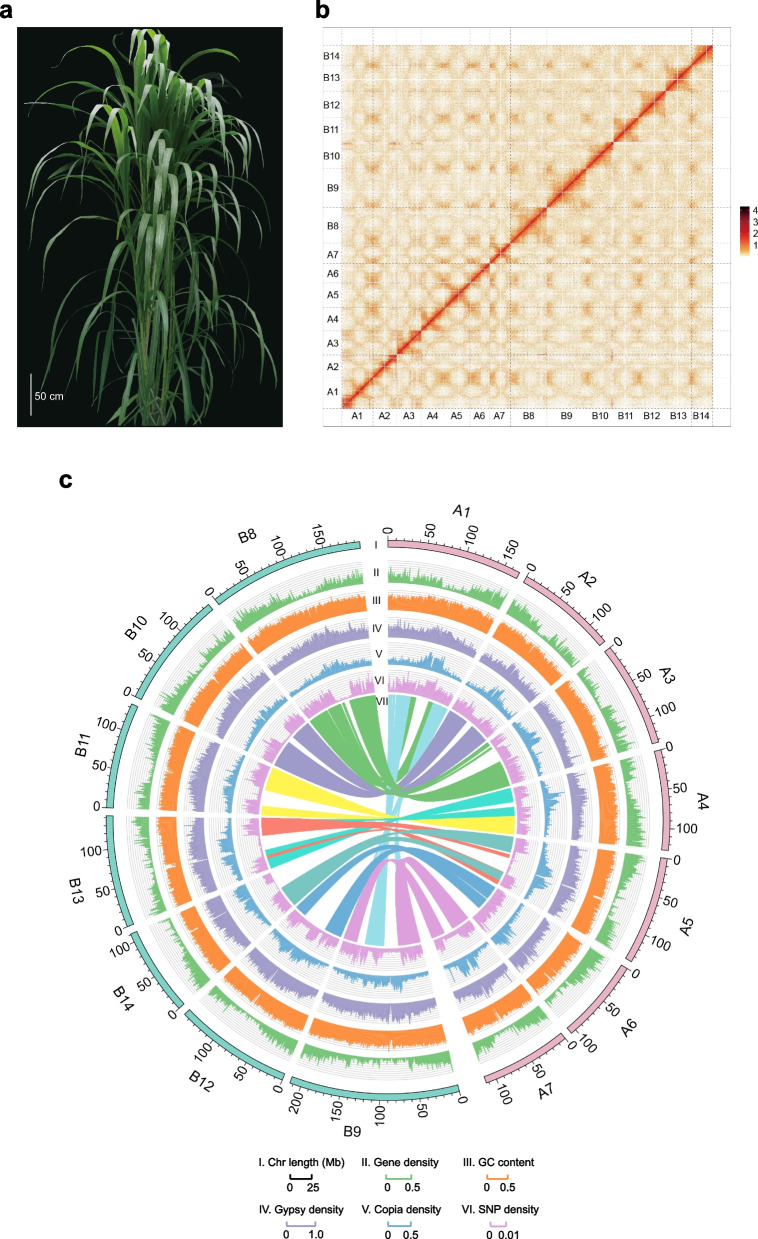
Overview of the *Pennisetum giganteum* genome assembly. **a** Morphology of *P. giganteum*. Scale bar = 50 cm. **b** Genome-wide Hi-C interaction map of *P. giganteum*. The heat map shows the intensity signal of the Hi-C chromosome interaction. **c** Circos representation of *P. giganteum* genome features. The outermost layer of blocks is a circular representation of the 14 pseudo-chromosomes, with scale mark at each 10 Mb (I). Density of protein-coding genes (II), GC content (III), LTR/*Copia* (IV), LTR/*Gypsy* (V), and genome-wide heterozygous single-nucleotide polymorphisms (SNPs) (VI) computed using a 100-kb non-overlapping window. The innermost track shows inter-chromosomal synteny relationship, with colored links that represent syntenic blocks between the two subgenomes

*P. giganteum* is an allotetraploid plant with many excellent characteristics of typical polyploid plants, such as huge size, high yield, and strong stress resistance (Fig. 1a). Previous studies have shown that polyploidization is an important driving force for increasing biomass and improving stress tolerance of plants during evolution [19–21]. However, the underlying mechanism of these traits in polyploid plants is still not fully elucidated. Besides, the nutrient content such as crude protein and cellulose was found to vary greatly in different growth stages of *P. giganteum*, but the underlying mechanism is also largely unexplored [8]. For example, the crude protein content in *P. giganteum* (height = 50 cm) at 4 weeks was 10.8%, but only 5.9% at 12 weeks [8]. Therefore, in-depth study of gene expression between the *P. giganteum* A and B subgenomes during different developmental stages will provide better insights into the potential mechanisms underlying biomass and nutrient content accumulation in polyploid plants.

So far, few transcriptome datasets on *P. giganteum* have been released; one reported the response of *P. giganteum* to chilling cold [22]. Only a few genome assemblies of *Pennisetum* species are available, such as pearl millet and elephant grass [23–25]. This lack of omics data has seriously hampered comprehensive evolutionary and comparative genomics studies of *Pennisetum* species. Therefore, to obtain more high-quality genome assemblies, it is necessary to expand the genetic resources of *Pennisetum* and elucidate the genetic basis of many important agronomic traits in genus *Pennisetum*. C4 plants generally have high photosynthetic efficiency, and the genetic basis of this is largely resolved [26, 27]. Additionally, many studies have focused on the evolution of C4 photosynthesis and the regulation of gene expression that are required for C4 photosynthesis previously [28–30]. However, the regulation and expression patterns of photosynthesis-related genes between subgenomes in polyploid C4 plants have rarely been reported.

Here, we present a high-quality genome assembly of the allotetraploid *P. giganteum* that was divided into A and B subgenomes. We examined the evolutionary relationship and the history of genomes across the Panicoideae lineages, and discovered the functional differentiation and homoeolog-biased expression between the A and B subgenomes by combining comparative genomics analysis with transcriptome meta-analysis. The results implied that the hybrid used the favorable copies of the genes and maintained their high expression. The comprehensive transcriptome analysis of *P. giganteum* leaf and stem at seven developmental stages revealed the genetic basis of biomass accumulation in this C4 plant*.* We also identified key candidate genes at each step of the C4 photosynthesis pathway that were consistently and highly expressed at all seven developmental stages. This genomic resource provides insights into the genetic basis of the huge biomass generated in polyploid plant species.

## Results

### High-quality genome assembly and separate A and B subgenomes of P. giganteum

We obtained a total of 65.25 Gb PacBio circular consensus sequencing (CCS) data and 284.94 Gb of high-throughput chromosome conformation capture (Hi-C) sequencing data and used them to generate a high-quality genome assembly of *P. giganteum* (Table S1). De novo assembly of CCS data yielded an initial contig-level assembly. We then used the Juicer and 3D-DNA algorithms to anchor and correct the assembled contigs by integrating the Hi-C data. The final assembly contained 14 chromosome pseudomolecules with 99.17% of the sequence anchored onto the chromosomes with contig N50 of 88.47 Mb. This assembly is one of the highest continuity grass genome assemblies currently available (Fig. 1b; Additional file 2: Figure S1; Table 1; Additional file 1: Tables S3 and S5). The total length of the assembly spanned 2.03 Gb, which is consistent with the estimated genome size from k-mer frequency analysis based on 115.01 Gb MGI-Seq short reads (Table 1; Additional file 2: Figure S2; Additional file 1: Tables S1–S3). Noticeably, four chromosomes consisted of one single contig, and the remaining 10 chromosomes harbored only 1–5 gaps (Additional file 1: Table S4), indicating the genome assembly had high contiguity. Benchmarking Universal Single-Copy Orthologs (BUSCO) evaluation showed that 98.50% of the genes identified in the *P. giganteum* genome were complete, and the average LTR assembly index score was 19.32 (Table 1; Additional file 1: Table S6), indicating that the genome assembly was close to the gold standard [31]. The average of RNA sequencing (RNA-seq) mapping rates was 96.09%, further confirming the completeness of the genome assembly (Additional file 1: Table S7). Additionally, the base accuracy evaluation by Illumina short reads indicated > 99.999% accuracy rate of the *P. giganteum* genome (Additional file 1: Table S8). Together, these results confirmed the high quality of our *P. giganteum* genome assembly.

**Table 1:** Statistics of the *Pennisetum giganteum* genome assembly and annotation

	***P. giganteum***
**Genome assembly**	
Estimated genome size (Gb)	1.90
Assembled genome size (Gb)	2.03
A subgenome size (Gb)	0.92
B subgenome size (Gb)	1.09
Number of contigs	83
Contig N50 (Mb)	88.47
Longest scaffold (Mb)	147.88
Chromosome numbers	14
Anchored rate (%)	99.17
GC content	47.24%
BUSCO	98.5%
LAI	19.32
**Genome annotation**	
Repeat region % of assembly	66.12%
Predicted gene models	85,313
Gene models in A subgenome	42,746
Gene models in B subgenome	42,567
Average gene sequence length (bp)	3042
Average exons per gene	4.29
Average exon length (bp)	258

Overall, 66.12% of *P. giganteum* genome sequence was identified as transposable elements, and long terminal repeat retrotransposons (LTR-RTs) were the most abundant class of repetitive sequences, accounting for 52.14% of the *P. giganteum* genome. Among the LTR-RTs, *Gypsy* elements (31.45%) were the most abundant, followed by *Copia* elements (11.50%) (Fig. 1c; Table 1; Additional file 1: Tables S9–S10). A total of 26,679 intact LTR-RTs were identified, including 13,312 *Gypsy* elements (49.90%) and 8216 *Copia* elements (30.80%) (Additional file 1: Table S11). A total of 85,765 high-confidence protein-coding genes were predicted by combing an evidence-based method and de novo prediction (Table 1; Additional file 1: Table S12); 95.89% of these genes were functionally annotated by homologous sequence searches and with Pfam protein domains by InterProScan (Table 1; Additional file 1: Table S13). The annotated gene set covered 99.40% of the complete BUSCO genes (Additional file 1: Table S6), indicating the completeness of the gene annotation. The average gene length and average exon number were 3042 bp and 4.29, respectively (Additional file 1: Table S12), which is consistent with those in the genomes of two other *Pennisetum* species, elephant grass (*Pennisetum purpureum*) and pearl millet (*Cenchrus americanus*) [24].

To divide *P. giganteum* genome into subgenomes, resequencing reads from *C. americanus* (2n = 14), potential donor of the A subgenome in the genus *Pennisetum* [5], were mapped onto the *P. giganteum* genome. The mapping rates of seven chromosomes were 4.67–30.05%, whereas those of the other seven chromosomes were < 2.47% (Additional file 2: Figure S3; Additional file 1: Table S14). We also identified and analyzed the frequencies of 13-mers enriched in the *P. giganteum* genome and found that the enriched 13-mer species divided the 14 chromosomes into two clear groups (Additional file 2: Figure S4; Additional file 1: Table S15). Phylogenetic analysis based on the alignment of single-copy genes of homologous chromosome pairs of *P. giganteum*, *C. americanus*, and *Setaria viridis* showed that the 14 chromosomes of *P. giganteum* again divided into two clear groups; the seven chromosomes in one of the groups were closer to *C. americanus* in phylogenetic distance than the seven chromosomes in the other group (Additional file 2: Figures S5–S7). Together these results indicate that the *P. giganteum* genome was successfully sorted into A and B subgenomes that contained seven chromosomes each (Fig. 1c).

### Comparative genomics and gene family evolution analysis

**Fig. 2:**
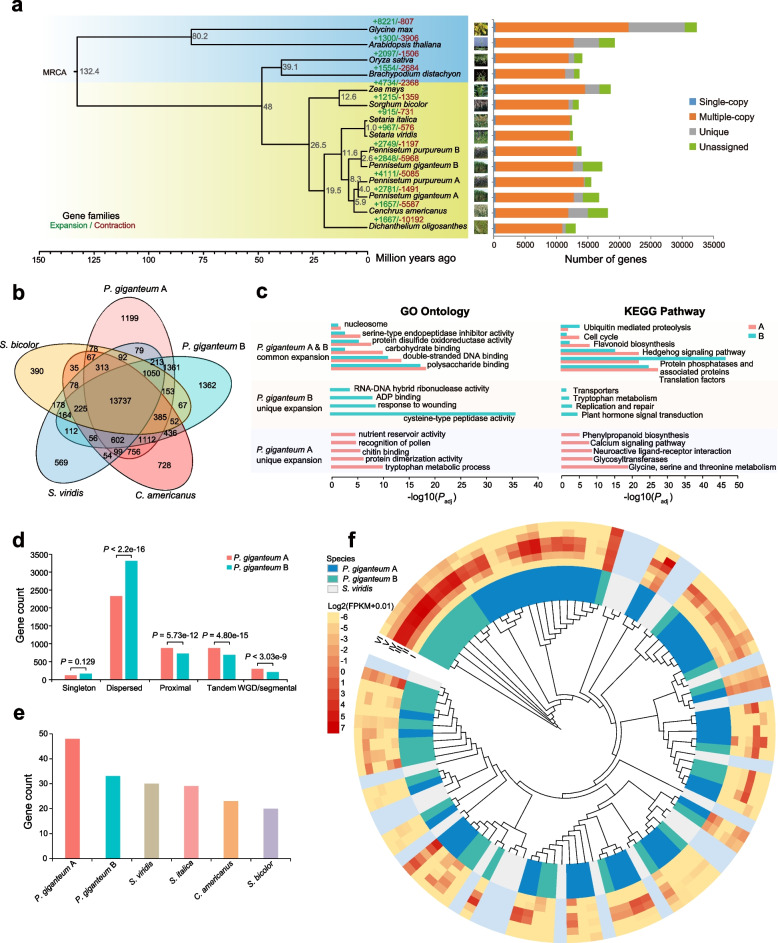
Comparative genomics analysis between *Pennisetum giganteum* and other grass species. **a** Phylogenetic relationship of *P. giganteum* with 11 other plant species. C3 species are shaded in blue; C4 species are shaded in yellow. Species divergence times were estimated using r8s based on one calibration time point obtained from the TimeTree website. Estimated divergence times are shown in gray. The number of expanded and contracted gene families obtained using CAFE based on the orthologous groups of each species assigned by OrthoFinder are given below the species names in green and red, respectively. Distribution of different categories of protein-coding genes across all the species is shown on the right. **b** Venn diagram of gene families among five species. A total of 13,737 common gene families were identified among these species, and the number of unique gene families is listed for each species. **c** Gene ontology (GO) and Kyoto Encyclopedia of Genes and Gnomes (KEGG) enrichment analysis of genes that were commonly and uniquely expanded in the A and B subgenomes of *P. giganteum*. **d** Classification of gene duplicates in the A and B subgenomes of *P. giganteum*. The origins of duplicated genes were categorized into five types: whole genome/segmental duplication, tandem duplication, proximal duplication, dispersed duplication, and singleton. Statistical significance was determined using Fisher’s exact test. **e** Distribution of genes encoding proteins containing the N-terminal domain of chalcone and stilbene synthases (CHS/STS) in two subgenomes of *P. giganteum* and four other related species. **f** Phylogeny of the chalcone synthase (CHS) gene family. The inner tracks show CHS genes identified in the A and B subgenomes of *P. giganteum* and *S. viridis*. The outer six tracks show the expression level of CHS genes in different tissues in the two subgenomes of *P. giganteum*. I, Apt; II, Int; III, Root; IV, Seedling below; V, Seedling above; VI, Stem. The log2(FPKM + 0.01) level is indicated by the intensity of the color. *S. viridis* is in gray because of the lack of expression data

To investigate the evolution of *P. giganteum*, we constructed a phylogenetic tree of 12 plant species (*P. giganteum* and *P. purpureum* were divided into two subgenomes) using 307 single-copy genes (Additional file 1: Table S17). Phylogenomic analysis showed that the A subgenome of *P. giganteum* and *C. americanus* originated from a common ancestor 5.9 million years ago (Mya), followed by the divergence of the A subgenome of *P. purpureum* and *P. giganteum* at 4.0 Mya (Fig. 2a). The B subgenome of *P. purpureum* and *P. giganteum* diverged more recently (approximately 2.6 Mya). Formation of the allotetraploid *P. giganteum* genome (AABB) occurred at approximately 8.3 Mya, at which time the two subgenomes of *P. giganteum* diverged from each other. Gene family expansion and contraction analysis showed that 2781 and 2848 gene families were expanded in the A and B subgenomes of *P. giganteum* after divergence from the common ancestor, respectively, and 901 of these gene families were unique to the *P. giganteum* genome (Fig. 2a; Additional file 1: Tables S16–S17). Most of the expanded gene families originated from dispersed duplication in both subgenomes, followed by proximal and tandem duplications (Fig. 2d). Notably, the percentage of dispersed duplication in the B subgenome (3310, 7.78%) was significantly higher than that in the A subgenome (2327, 5.44%) (*P* < 2.2 × 10^−16^, one-tailed Fisher’s exact test), while the percentages of tandem (*P* = 4.8 × 10^−15^), proximal (*P* = 5.73 × 10^−12^), WGD/segmental duplication (*P* = 3.03 × 10^−9^) in the B subgenome were significantly lower than those in the A subgenome.

Gene ontology (GO) and Kyoto Encyclopedia of Genes and Genomes (KEGG) functional enrichment analysis showed that the rapidly expanded gene families in the A subgenome (539 gene families) and B (647 gene families) subgenomes were commonly enriched in nucleosome (GO: 0005509; A: 15/1710, Benjamini-Hochberg (BH)-adjusted *P* = 9.70 × 10^−3^; B: 13/1608, BH-adjusted *P* = 4.25 × 10^−2^), polysaccharide binding (GO:0030247; A:54/1710, BH-adjusted *P* = 4.23 × 10^−19^; B: 47/1608, BH-adjusted *P* = 5.00 × 10^−18^), and translation factors (A: K15032, 29/399, BH-adjusted *P* = 2.36 × 10^−28^; B: K09419, 5/271, BH-adjusted *P* = 1.84 × 10^−5^), implying these gene families were involved in maintaining basic life activities (Fig. 2c; Additional file 1: Tables S18–S19). The expanded gene families in the A subgenome were uniquely enriched in recognition of pollen (GO:0048544, 23/1710, BH-adjusted *P* = 1.52 × 10^−5^), chitin binding (GO:0008061, 11/1710, BH-adjusted *P* = 2.11 × 10^−6^), tryptophan synthase activity (GO:0004834, 16/1710, BH-adjusted *P* = 8.79 × 10^−13^), nutrient reservoir activity (GO:0045735, 18/1710, BH-adjusted *P* = 1.95 × 10^−5^), and protein dimerization activity (GO:0046983, 61/1710, BH-adjusted *P* = 5.10 × 10^−7^) (Fig. 2c; Additional file 1: Table S18). The KEGG pathway analysis showed that the expanded gene families in the A subgenome were uniquely involved in phenylpropanoid biosynthesis (K00083, 6/399, BH-adjusted *P* = 7.89 × 10^−6^), calcium signaling pathway (K04150, 27/399, BH-adjusted *P* = 1.34 × 10^−8^), and glycosyltransferases (K08237, 8/399, BH-adjusted *P* = 1.59 × 10^−9^) (Fig. 2c; Additional file 1: Table S19). The expanded gene families in the B subgenome were uniquely enriched in nucleic (GO:0000786, 13/1608, BH-adjusted *P* = 1.33 × 10^−2^), ADP binding (GO:0043531, 70/1608, BH-adjusted *P* = 4.25 × 10^−2^), and response to wounding (GO:0009611, 15/1608, BH-adjusted *P* = 1.68 × 10^−9^) (Fig. 2c; Additional file 1: Table S18). The KEGG pathway analysis showed that the expanded gene families in the B subgenome were uniquely involved in plant hormone signal transduction (K13464, 6/271, BH-adjusted *P* = 2.99 × 10^−5^), replication and repair (K07497, 4/271, BH-adjusted *P* = 1.07 × 10^−3^), and transporters (K24193, 3/271, BH-adjusted *P* = 4.63 × 10^−2^) (Fig. 2c; Additional file 1: Table S19). Together, the functional enrichment results suggested that the expanded gene families may improve the material synthesis capacity and environmental adaptability of *P. giganteum*, which is consistent with its high tolerance of abiotic stress and significantly high biomass. The functional differentiation of the expanded gene families between the A and B subgenomes of *P. giganteum* indicated that the A subgenome was related mainly to nutrient synthesis whereas the B subgenome was related mainly to abiotic stress tolerance.

Interestingly, gene families related to flavonoid biosynthesis were significantly expanded in the *P. giganteum* genome (Fig. 2c). Previous studies have demonstrated that flavonoids played important roles in the growth, development, and disease resistance of plants [32]. Specifically, we found that the chalcone and stilbene synthase (CHS/STS) gene family was significantly expanded in *P. giganteum* (A: 48, B: 33) compared with its presence in closely related species (*Sorghum bicolor*: 30, *C. americanus*: 29, *Setaria viridis*: 23, *Setaria italica*: 20) (Fig. 2e). Additionally, the copy number and expressed gene number in the CHS/STS family were much higher in the A subgenome (48) than they were in the B subgenome (33) (Fig. 2e, f; Additional file 2: Figure S8), implying that the A subgenome contributed more to flavonoid biosynthesis than the B subgenome, further confirming functional differentiation between the subgenomes in *P. giganteum*.

We compared the gene families in four typical C4 species (*P. giganteum*, *C. americanus*, *S. viridis*, and *S. bicolor*). Functional enrichment analysis found that the highly conserved genes among these C4 plants were significantly enriched in substance synthesis and stress resistance in the GO biological process category (Additional file 2: Figure S9; Additional file 1: Tables S20–S21), which may be related to features of these C4 plants. The unique gene families detected in the A and B subgenomes of *P. giganteum* by comparison were mainly enriched in DNA integration, photosynthesis, gene silencing by RNA, negative regulation of translation, defense response, and response to wounding (Additional file 2: Figure S10; Additional file 1: Tables S22–S24), which implied that *P. giganteum* evolved to better cope with biotic and abiotic stress environment compared with the other C4 plants. Furthermore, 2292 and 2608 unique genes in the A and B subgenomes were identified by comparing the two subgenomes. The unique genes in the A subgenome were involved mainly in substance metabolism and stress response, whereas the unique genes in the B subgenome were associated mainly with material biosynthesis (Additional file 2: Figure S11; Additional file 1: Table S25). We also found that there were significant differences in the expression levels of conserved and unique genes in *P. giganteum* (Additional file 2: Figure S12).

### Comparative genomic analysis between P. giganteum and its closely related species P. purpureum

**Fig. 3:**
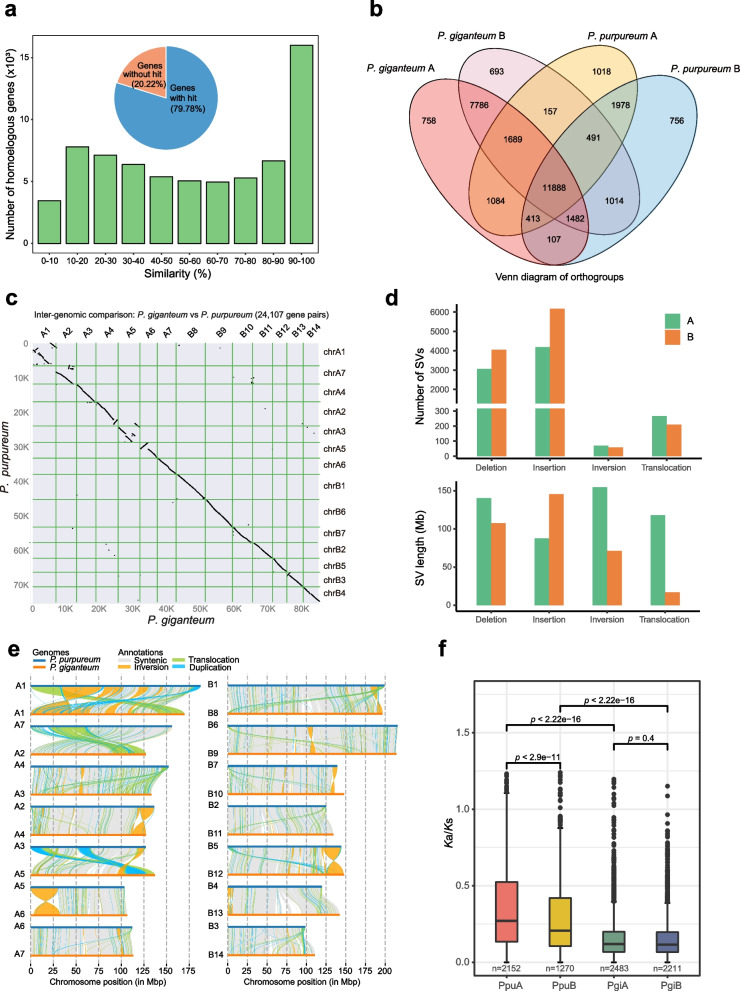
Comparative genomics analysis between *P. giganteum* and *P. purpureum*. **a** Histogram showing the distribution of protein sequence similarity between *P. giganteum* and *P. purpureum*. *x*-axis represents the intervals of percent similarity, and *y*-axis represents the number of homologous gene pairs. The inset pie chart represents the number and percentage of *P. giganteum* genes having significant BLAST hits against *P. purpureum* genes. **b** Venn diagram showing the common and unique orthogroups (OGs) identified in subgenomes of *P. giganteum* and *P. purpureum*. In total, 31,314 OGs were assigned by OrthoFinder, of which 11,888 were commonly detected in four subgenomes. **c** Genome-wide synteny analysis between *P. giganteum* and *P. purpureum* using JCVI. A total of 24,107 syntenic gene pairs were obtained through inter-genomic comparison. Nearly all the chromosomes exhibited 1:1 correspondence between two *Pennisetum* species. However, many genome rearrangements were observed between two species. **d** Statistics of structural variations (SVs) identified between *P. giganteum* and *P. purpureum* through SyRI analysis. SVs are categorized into four major types, including deletion, insertion, inversion, and translocation. The upper panel denotes the number of four types of SVs in the A and B subgenomes, and the lower panel represents the cumulative length of four types of SVs in two subgenomes. The A subgenome is shaded in green, and the B subgenome is highlighted in orange. The cumulative length of different types of SVs was calculated with the *P. giganteum* as the reference. **e** Visualization of genome structural rearrangements using the visualization tool *plotsr*. The left panel denotes the result of the A subgenome, and the right panel represents the result of the B subgenome. Each graph was composed of a pair of homologous chromosomes. The color of the links denotes the type of annotations: (1) syntenic regions are shaded in gray; (2) inversions are highlighted in orange; (3) translocations are colored in green; and (4) duplications are shaded in blue. **f** Box and whisker plot showing the distribution of nonsynonymous to synonymous substitution ratios (*K*a/*K*s) in two subgenomes of *P. giganteum* and *P. purpureum*. The upper and lower vertical extending lines denote the most extreme values within 1.5 interquartile range of the 75th and 25th percentile of each group, respectively. The dots above the upper line denote the outliers. The number of gene pairs used for *K*a/*K*s calculation was listed below the boxplot for each subgenome. Statistical significance was determined using two-sided Wilcoxon rank-sum test

Considering the high similarity in morphological characteristics and biological traits between *P. giganteum* and *P. purpureum*, we undertook a detailed comparison analysis between two species to explore their genomic difference. Firstly, sequence similarity analysis showed that 20.22% (17,251/85,313) of *P. giganteum* proteins had no BLAST hit against *P. purpureum* proteins (Fig. 3a), and 23.50, 49.50, and 27.00% of homologous gene pairs showed low (identity < 30%), moderate (30% ≤ identity < 90%), and high (identity ≥ 90%) sequence similarity, respectively (Fig. 3a), suggesting that there exists substantial difference in sequence between two species. Secondly, the proteins in two subgenomes of *P. giganteum* and *P. purpureum* were clustered, and a total of 31,314 orthogroups (OGs) were assigned. Among them, 11,888 (37.96%) OGs were shared by four subgenomes, while a substantial proportion of OGs were identified only in either species (Fig. 3b), suggesting the large differences in gene family between two species. For example, there were more copies of cellulose synthase-related genes (*P. giganteum*: 73; *P. purpureum*: 63) and C4 photosynthesis-related genes (*P. giganteum*: 78; *P. purpureum*: 65) in *P. giganteum* compared with their presence in *P. purpureum*. Further analysis of different types of duplicated genes showed that there were great differences in percentage between *P. giganteum* and *P. purpureum* (Additional file 2: Figure S13). Thirdly, genome-wide synteny analysis showed that almost all the chromosomes displayed 1:1 correspondence between two species (Fig. 3c). However, extensive genome rearrangements were detected, especially for chromosomes PgiA1, PgiA2, PgiA5, PgiA6, and PgiB12, suggesting the obvious changes in chromosome structure between two species during evolution. Fourth, the structural variations (SVs) between two genome assemblies were identified, yielding a total of 18,062 large SVs (> 50 bp) (Fig. 3e). The results showed that deletion (7108) and insertion (10,350) are top two most abundant types of SVs, followed by translocation (476) and inversion (128) (Fig. 3d, upper panel). As with the SV length, deletion, insertion, inversion, and translocation events spanned 248.28, 233.38, 226.09, and 135.09 Mb, respectively, suggesting that SVs involved a large fraction of genomic region (40.68%) (Fig. 3d, lower panel). Notably, several large inversions were observed in PgiA1 (0.28–45.26 Mb; 45.45–54.12 Mb; 54.14–61.34 Mb; 82.02–92.76 Mb), PgiA4 (112.85–127.17 Mb), PgiA6 (3.50–32.64 Mb), and PgiB12 (126.45–147.19 Mb) (Fig. 3e). Additionally, we made a comparison of SV count and length with four other groups of plants and found that the count and length of SVs varied greatly among these groups (Additional file 1: Table S26). In terms of insertion, we observed the longest average insertion length between *P. purpureum* and *C. americanus*, followed by *P. purpureum* and *P. giganteum* (Additional file 1: Table S26). These results indicated the occurrence of extensive genome rearrangements and great divergences between *P. giganteum* and *P. purpureum*. Fifth, we calculated the ratios of nonsynonymous substitution rate to synonymous substitution rate (*K*a/*K*s) for each subgenome of two species. The results showed that both sets of subgenomes exhibited significant differences in evolutionary rate between two species (A: *P* < 2.22 × 10^−16^; B: *P* < 2.22 × 10^−16^, Wilcoxon rank-sum test) (Fig. 3f). Interestingly, *K*a/*K*s ratios were significantly different between the A and B subgenomes of *P. purpureum* (*P* < 2.9 × 10^−11^), while no significant difference was observed between two subgenomes of *P. giganteum* (*P* = 0.4) (Fig. 3f). Altogether, the above evidences confirmed the substantial differences between *P. giganteum* and *P. purpureum*, suggesting that *P. giganteum* is a different species from *P. purpureum* despite their high similarity in phenotype.

### Genome evolution analysis of the A and B subgenomes

**Fig. 4:**
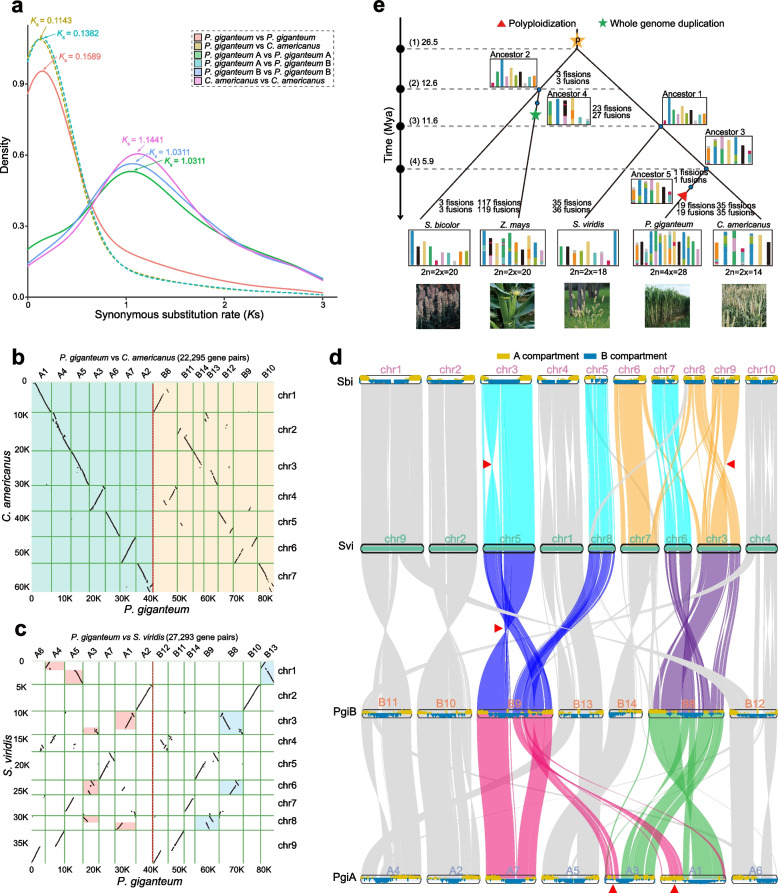
Genome evolutionary history of *P*ennisetum *giganteum*. **a** Distribution of inter- or intra-species synonymous substitution rates (*K*s) indicates whole-genome duplication (WGD) events and species divergence time. The different colored curves indicate the *K*s distribution of syntelogues between two species or subgenomes of the same species. **b** Genome-wide synteny analysis between *P. giganteum* and *C. americanus*. **c** Genome-wide synteny analysis between *P. giganteum* and *S. viridis*. **d** Chromosome number evolution of grass species detected by genome-wide comparison across two subgenomes of *P. giganteum* and two other species. The syntenic blocks among *S. bicolor*, *S. viridis*, and the A and B subgenomes of *P. giganteum* were generated using the JCVI utility (Python version of MCScan). **e** Reconstructed ancestral grass karyotype based on five extant grass species, namely *P. giganteum*, *S. bicolor*, *Z. mays*, *S. viridis*, and *C. americanus*, using the IAGS pipeline. The inferred karyotypes of five ancestry nodes and the five representative grass species are displayed together with a schematic species tree that shows the evolutionary history of these grass species. The red triangle indicates the polyploidization event and the green pentagram indicates the WGD event. The timeline (leftmost) indicates species divergence times in Mya. Chromosome numbers of the five grass species are given below the karyotypes of each species. Divergence times of (1) *S. bicolor* and *C. americanus,* (2) *S. bicolor* and *Z. mays,* (3) *S. viridis* and *P. giganteum*, and (4) *P. giganteum* and *C. americanus*

A total of 22,295 pairs of syntenic genes were identified between *C*. *americanus* and *P. giganteum* (A: 21,336; B: 19,490) (Fig. 4b; Additional file 2: Figure S14a, b). The A subgenome of *P. giganteum* was strongly collinear with *C*. *americanus*, and their chromosomes showed an almost 1:1 syntenic relationship (Fig. 4b; Additional file 2: Figure S14c). Conversely, many large-scale chromosomal rearrangements were identified between the B subgenome and *C*. *americanus* (Fig. 4b). Interestingly, we found that the B subgenome was highly syntenic with *S. viridis*, except for several rearrangements (Fig. 4c), and more highly conserved genes were found between the B subgenome and *S. viridis* than were found between the B subgenome and *C*. *americanus* (Additional file 2: Figure S15; Additional file 1: Table S27)*.* Together, our results show that the A subgenome was evolutionarily closer to *C*. *americanus*, and the B subgenome was evolutionarily closer to *S. viridis* indicating it had a closer relationship to the ancestral status than the A subgenome had.

The synteny analysis between the A and B subgenomes of *P. giganteum* identified numerous genome rearrangements between them (Fig. 4d). Chromosome B8 contained the major parts of chromosomes A1 and A3, and the remaining parts of the A1 and A3 chromosomes were inserted into the middle part of chromosome A7 to create chromosome B9. Chromosome A4 contributed to the major part of chromosome B11 and the left part of chromosome B13, and the remaining part of chromosome B11 showed synteny with chromosome A6. Chromosome A5 was split into the major part of chromosome B14 and the right part of chromosome B13 (Fig. 4d; Additional file 2: Figure S16). Interestingly, these chromosomal rearrangement events tended to occur frequently in candidate pericentromeric regions of the *P. giganteum* chromosomes (Additional file 2: Figures S17-S18). Previous studies found that the A and B compartments are active and inactive chromatin regions, respectively [33, 34]. Thus, the switching of A/B compartment status might influence gene expression in corresponding genomic regions. To examine the effect of genomic rearrangement on the switching of A/B compartment status, we investigated the switching patterns of chromatin A/B compartment for syntenic blocks between the two subgenomes of *P. giganteum*. We found that most 3D chromatin A/B compartments were stable between the A and B subgenomes, with stable A/B compartment ratios of 57.24–83.42% (Additional file 1: Table S28), indicating a highly conserved chromatin spatial structure. However, we also found extensive compartment status switching from A to B or B to A between the two subgenomes (Additional file 2: Figure S18a). Further, we found that the rearranged genomic regions (41.84%) were more likely to undergo A/B compartment switching compared with the non-rearranged genomic regions (27.04%) (*P* < 2.2 × 10^−16^, one-tailed Fisher’s exact test) (Additional file 2: Figure S18b), suggesting that genome rearrangement may be an important factor that contributes to changes in gene regulation and expression. Additionally, an inter-genomic comparison identified eight inversions of large fragments (> 1 Mb) between the A and B subgenomes that were validated by the Hi-C contact map (Additional file 2: Figure S16). Together, these findings show that many genome rearrangements occurred between the A and B subgenomes of *P. giganteum*. These rearrangements may be an important driving force of species evolution.

We reconstructed the ancestral karyotypes of *P. giganteum*, *S. viridis*, *S. bicolor*, *C. americanus*, and *Zea mays* (Fig. 4d, e). The ancestral karyotypes showed that the common ancestor of *P. giganteum* and *C. americanus* had seven chromosomes. Prior to polyploidization of *P. giganteum*, ancestor 5 split from ancestor 3 through one fission and one fusion. The allotetraploid *P. giganteum* emerged after ancestor 5 underwent hybridization and 19 fission and 19 fusion events. The fewest rearrangements were recorded in *S. bicolor* relative to ancestor 2 (three fissions and three fusions), and the most rearrangements were detected in *Z. mays* relative to ancestor 4 (117 fissions and 119 fusions), partly because of the lineage-specific whole-genome duplication (WGD) event that occurred in *Z. mays*. Specifically, a large segmental inversion was identified between the front of chromosome 3 of *S. bicolor* and chromosome 5 of *S. viridis*, and this genomic region of *S. viridis* was completely collinear with chromosomes B9 and A7 of *P. giganteum*. Thus, we speculated that this inversion occurred only in the *S. bicolor* genome and that the ancestral state was likely maintained in *P. giganteum* and *S. viridis*. The B8 chromosome of *P. giganteum* was formed by the insertion of chromosome 6 of *S. viridis* into the near-middle part of chromosome 3 of *S. viridis*, and chromosome 7 of *S. bicolor* was completely collinear with chromosome 6 of *S. viridis*, suggesting that *S. bicolor* and *S. viridis* harbored the ancestral state of these regions. The reconstructed ancestral karyotypes provide a global view of genome rearrangements across these species, further supporting chromosomal rearrangement as an important driving force of genome evolution and speciation.

### Analysis of differentially expressed homoeologous genes between the A and B subgenomes

**Fig. 5:**
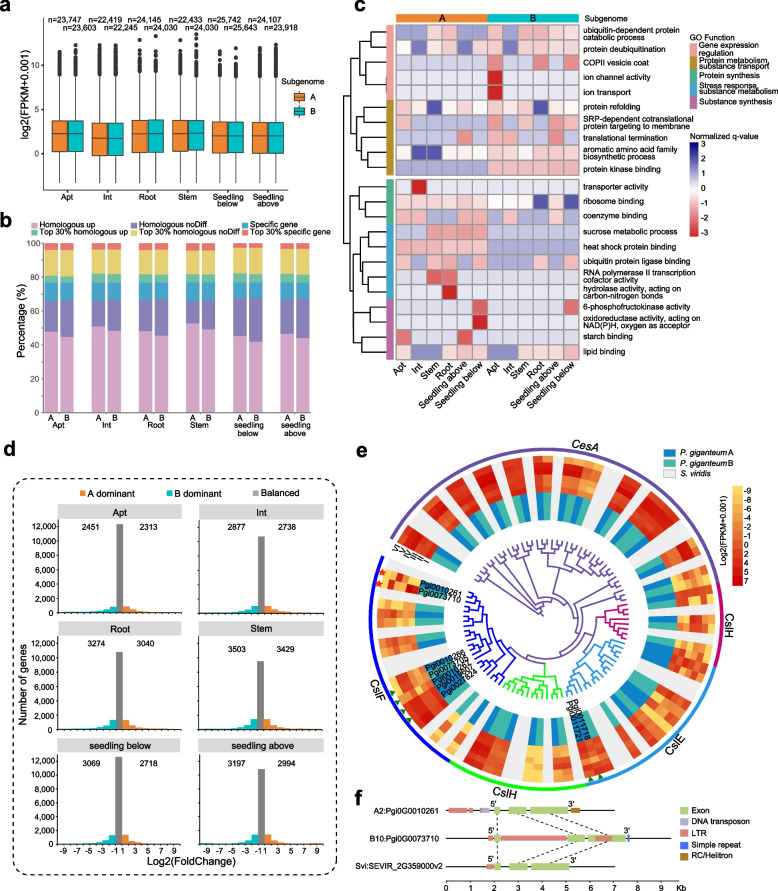
Divergence of gene expression and molecular functions between the two subgenomes of *Pennisetum giganteum*. **a** Boxplot showing expression levels of all the expressed genes (FPKM ≥ 0.1) across different tissues in the A and B subgenomes of *P. giganteum*. The numbers of expressed genes in the different tissues of subgenomes are given on the top. **b** Histogram showing distinct categories of all the expressed and highly expressed genes across different tissues in the A and B subgenomes. **c** Gene ontology (GO) enrichment of highly expressed genes in different tissues of the two subgenomes. Highly expressed genes in subgenome A were enriched in GO terms mainly related to protein synthesis, stress response and substance metabolism, and substance synthesis. Highly expressed genes in subgenome B were enriched in GO terms mainly associated with gene expression regulation, protein metabolism, and substance transport. **d** Histograms of genome-wide expression of homoeologous genes in six different tissues of *P. giganteum*. The numbers of dominant genes in the A and B subgenomes are displayed for each tissue. **e** Phylogeny of cellulose synthase-related gene family. The outermost tracks show the five gene types: *CesA*, *CslD*, *CslE*, *CslF*, and *CslH*. Red stars indicate the candidate cellulose synthase genes; Green triangles indicate multi-copy genes in the A and B subgenomes. The inner tracks show the cellulose synthase genes identified in the A and B subgenomes and in *S. viridis*. The middle tracks show the expression level of cellulose synthase-related genes across different tissues in the two subgenomes. I, Apt; II, Int; III, Root; IV, Seedling below; V, Seedling above; VI, Stem. The log2(FPKM + 0.001) level is indicated by the intensity of the color. *S. viridis* is in gray because of the lack of expression data. **f** Gene structures of homologous cellulose synthase-related genes spanning the gene body and 2-kb regions upstream and downstream in the A and B subgenomes and in *S. viridis*

To investigate the gene expression patterns between the subgenomes in *P. giganteum*, we performed a comprehensive transcriptome data analysis of six *P. giganteum* tissues, apical tip, inverted third tip, root, stem, and aboveground and underground seedling parts. The mapping ratios of the two subgenomes and gene regions were similar for each tissue (A: 49.09–50.63%, B: 49.37–50.91%) with difference of only 0.55–1.82% (Additional file 1: Table S29). The number of genes expressed in the B subgenome (22,245–25,643) was similar to the number in the A subgenome (22,419–25,742) for each tissue (Fig. 5a). Gene expression analysis showed that most of the expressed genes (87–89%) in the different tissues were homoeologous genes between the subgenomes, and the remaining 11–13% were subgenome-specific genes (Fig. 5b). These findings implied that there was no significant subgenome dominance in gene expression in *P. giganteum*. However, the differential expression analysis between paired tissues revealed a clear tissue-specific pattern of gene expression in *P. giganteum* (Additional file 1: Table S31; Additional file 2: Figures S19–S21). Functional enrichment analysis showed that the highly expressed genes (top 30% based on the FPKM) in the A subgenome were related to protein synthesis (GO:0005840, BH-adjusted *P* = 7.17 × 10^−3^–2.06 × 10^−2^), whereas the highly expressed genes in the B subgenome were enriched in protein metabolism (GO:0019752, BH-adjusted *P* = 2.34 × 10^−2^–6.70 × 10^−2^), substance transportation (GO:0030127, BH-adjusted *P* = 1.06 × 10^−2^–1.69 × 10^−2^), and gene expression regulation (GO:0003723, BH-adjusted *P* = 1.08 × 10^−2^–4.26 × 10^−2^) (Fig. 5c; Additional file 1: Table S30).

The differential expression of homoeologous genes is considered to be an important mechanism of heterosis in polyploid species [35]. A total of 21,248 pairs of homologous genes were identified between the A and B subgenomes. The homoeologous genes that showed significantly biased expression between the two subgenomes were defined as homoeologous differentially expressed genes (HDEGs). The homoeologous genes that were expressed only in one subgenome were defined as homoeolog-specific expressed genes (HSEGs). Notably, most of the expressed homoeologous gene pairs exhibited balanced expression (57.86–72.11%) between the two subgenomes for each tissue (Fig. 5d; Additional file 1: Table S32), further supporting the idea that there was no significant subgenome dominance in gene expression in *P. giganteum*. However, 22.42–32.62% of homoeologous genes displayed expression bias between the two subgenomes, and the genes with higher expression levels were defined as A or B dominant genes. We found that there were slightly more B dominant homoeologous genes (2451–3503) than A dominant homoeologous genes (2313–3429) for each tissue (Fig. 5d; Additional file 1: Table S32). Interestingly, 637 HDEGs were consistently dominant in the A subgenome (Additional file 2: Figure S22a), and 831 HDEGs were consistently dominant in the B subgenome across all tissues (Additional file 2: Figure S22b). The enriched functions of the HDEGs in different tissues varied remarkably between the two subgenomes (Additional file 2: Figure S22c). For example, the HDEGs in apical leaf tip and inverted third tip were involved mainly in material hydrolysis (GO:0015914, BH-adjusted *P* = 3.33 × 10^−2^–4.28 × 10^−2^) in the A subgenome, while the B subgenome contributed to lipid metabolic process (GO:0006629, BH-adjusted *P* = 1.07 × 10^−2^–1.67 × 10^−2^), providing sufficient energy for plant growth (Additional file 2: Figure S22c). Intriguingly, more HSEGs were identified in the A subgenome (445–548) than in the B subgenome (286–455) for most tissues (Additional file 1: Table S3). These HSEGs were divided into two groups according to their expression patterns among different tissues: (1) consistent group in which the HSEGs were uniquely expressed in the same subgenome in all tissues (A: 64, B: 102) (Additional file 2: Figure S23a, b) and (2) discordant group in which the HSEGs were uniquely expressed in only one subgenome in some tissues (A: 1376, B: 1228) (Additional file 2: Figure S23c). This distinction may have important implications for the formation of allotetraploid advantage. Overall, the genome-wide expression analysis results indicated that gene expression was balanced between the two subgenomes. Nonetheless, many homoeologous genes showed expression bias between the two subgenomes among different tissues, implying that polyploidization can enable the use of favorable copies of genes, which are then expressed at high levels, strongly suggesting functional differentiation between the two subgenomes of *P. giganteum*.

*P. giganteum* is a cellulose-rich tetraploid plant, and unsurprisingly, we found that there were many more members of *Ces* and *Csl* gene families in *P. giganteum* (73, A: 39, B: 34) than there were in *Arabidopsis thaliana* (40), *Oryza sativa* (43), and *S. viridis* (32) (Fig. 5e; Additional file 1: Table S34), implying that tetraploidization may have contributed to the high content of cellulose in *P. giganteum*. Transcriptome analysis showed that the cellulose synthesis genes were highly expressed in the stem and leaf of *P. giganteum* (Fig. 5e; Additional file 1: Table S35), indicating their important contribution to cellulose synthesis in these tissues. The phylogenetic analysis identified 30 homologous pairs of cellulose synthase genes between the two subgenomes; however, their expression patterns in the A and B subgenomes were significantly different among tissues (Fig. 5e; Additional file 1: Table S36). There were 28 and 23 genes that were expressed in all tissues of the A and B subgenomes, respectively. Additionally, 8 ~ 18 pairs of genes related to cellulose synthase were differentially expressed between two subgenomes in different tissues. For example, *CesA1*, *CesA6*, *CesA8*, and *CslF* showed sustained higher expression levels in the A subgenome than they did in the B subgenome across different tissues, and many cellulose synthase-like genes showed higher expression in the B subgenome than their homologs did in the A subgenome (Fig. 5e; Additional file 1: Table S37). Gene structure comparison of the homoeologous genes showed that the LTR insertions may be the main force for the significant differences in expression of the cellulose synthase-related genes and their subsequent functional differentiation (Additional file 2: Figure S24). A pair of *CslF* homologs (*Pgi0G0010261* and *Pgi0G0073710*) in the two subgenomes showed significant differential expression among different tissues, and further analysis showed that two LTRs were inserted into the gene region of *Pgi0G0073710* (Fig. 5f), which might be related to the differential expression of *CslF* between the subgenomes. Together, these results indicate that the A subgenome, which has more copy numbers and higher expression levels of cellulose synthase-related genes, may contribute more to the highly efficient cellulose biosynthesis for biomass accumulation than the B subgenome does.

### Strengthened cellulose synthesis and C4 photosynthesis in P. giganteum

**Fig. 6:**
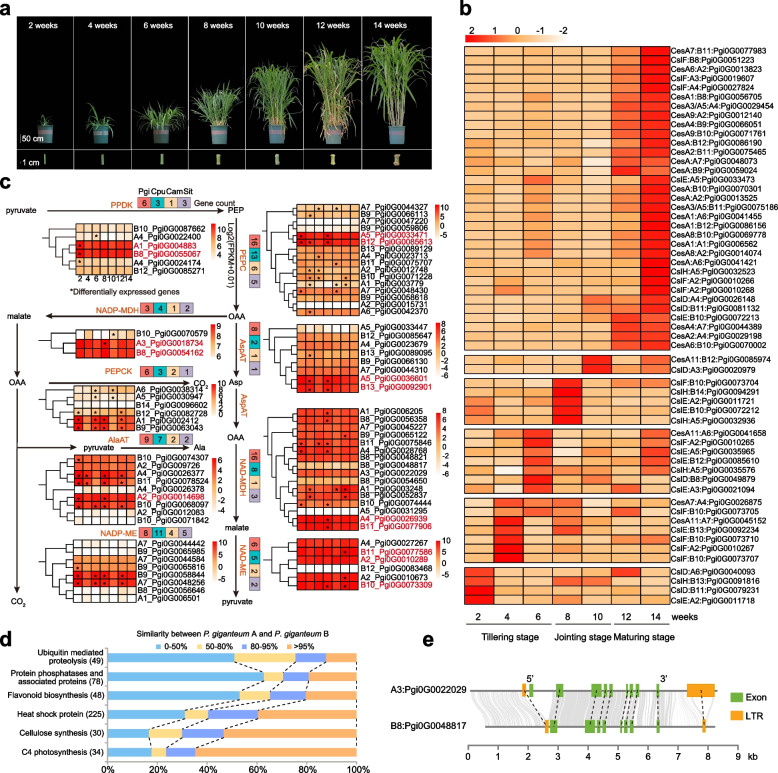
Highly efficient C4 photosynthesis pathway and cellulose synthesis contribute to the high biomass of *Pennisetum giganteum*. **a** Morphological characteristics of the whole plant and stem of *P. giganteum* at seven different periods, including three developing stages (tillering stage at 2, 4, 6 weeks, jointing stage at 8, 10 weeks, and maturing stage at 12, 14 weeks). Scale bars in the upper and lower panels correspond to 50 and 1 cm, respectively. **b** Heatmap of the expression of cellulose synthase-related genes that were expressed at, at least, one time point in leaves. **c** Simplified representation of the C4 photosynthesis pathway showing gene expression patterns of C4 pathway-related genes in leaf. PEPC, phosphoenolpyruvate carboxylase; NADP-ME, NADP-dependent malic enzyme; NAD-MDH, NAD-dependent malate dehydrogenase; AlaAT, alanine aminotransferase; NAD-ME, NAD-dependent malic enzyme; NADP-MDH, NADP-dependent malate dehydrogenase; PEPCK, phosphoenolpyruvate carboxykinase; PPDK, pyruvate/orthophosphate dikinase; AspAT, aspartate aminotransferase. The expression levels of C4 pathway-related genes in leaf are colored according to the log2(FPKM + 0.01) values across seven periods. Red indicates candidate C4 pathway-related genes that were highly expressed in seven periods; asterisks indicate genes that were differentially expressed at different developmental stages. The number of genes in each gene family is shown for four closely related species, *P. giganteum*, *P. purpureum*, *C. americanus*, and *S. italica*. **d** Similarity analysis of homologous genes in the A and B subgenomes of *P. giganteum*. **e** Gene structures of a pair of homologous NAD-MDH genes (*Pgi0G0022029* and *Pgi0G0048817*) spanning the gene body and 2-kb regions upstream and downstream in the A and B subgenomes. Both genes shared high similarity and all the exons had one-to-one relationships, except for *Pgi0G0022029*

*P. giganteum* is a typical polyploid and C4 plant that is well known for its rapid growth and huge biomass production. To discover the genetic basis of biomass accumulation of *P. giganteum*, we collected 42 transcriptome samples of leaf and stem at seven different developmental periods, including three developing stages, tillering, jointing, and maturing (Fig. 6a). The transcriptome analysis identified that a 20,517 and 26,596 differentially expressed genes (DEGs) among the different developmental periods of leaves and stems, respectively (Additional file 2: Figures S25–S28). Interestingly, the GO enrichment of these DEGs showed that the genes were significantly correlated with the growth and development requirements of each developmental stage in leaves and stems (Additional file 1: Tables S38–S40). Especially in leaves, the GO enrichment of DEGs among the different developmental stages showed that the highly expressed genes at the tillering stage were enriched mainly in cell proliferation (GO:2,001,109, BH-adjusted *P* = 1.24 × 10^−2^–4.08 × 10^−2^) and growth factor activity (GO:0008083, BH-adjusted *P* = 1.24 × 10^−2^–3.23 × 10^−2^), which mainly promoted the tillering of plants. At the jointing stage, the highly expressed genes were enriched mainly in photosynthesis (GO:0019684, BH-adjusted *P* = 1.02 × 10^−12^–2.58 × 10^−6^), cellulose biosynthesis (GO:2,001,006, BH-adjusted *P* = 3.62 × 10^−5^–4.28 × 10^−2^), material transport (GO:2,000,880, BH-adjusted *P* = 2.42 × 10^−7^–7.10 × 10^−3^), cytokinin (GO:0080038, BH-adjusted *P* = 3.33 × 10^−2^–4.28 × 10^−2^) and xyloglucan metabolic process (GO:0010411, BH-adjusted *P* = 1.69 × 10^−2^–3.74 × 10^−2^), which contribute mainly to plant growth and metabolite biosynthesis to meet the need of rapid growth. At the maturing stage when plant development had slowed down, the highly expressed genes were enriched mainly in metabolism-phenylalanine catabolic process (GO:0006559, BH-adjusted *P* = 3.58 × 10^−4^) and cyclin-dependent protein serine/threonine kinase inhibitor activity (GO:1,904,993, BH-adjusted *P* = 3.98 × 10^−2^) (Additional file 1: Table S39).

The transcriptome analysis showed that the cellulose synthase-related genes were significantly differentially expressed among different developmental stages in leaves (Fig. 6b; Additional file 2: Figure S29) and stems (Additional file 2: Figure S30). In leaves, five *CslE*, five *CslF*, and three *CslD* genes were highly expressed at the tillering stage; two *CslE* and two *CslH* genes were highly expressed at the jointing stage; and 23 *CesA* and five *CslF* genes were highly expressed at the maturing stage. In stems, 15 *CesA*, five *CslD* and six *CslF* genes were highly expressed at the tillering stage to promote primary wall synthesis and seedling growth; and eight *CslE*, eight *CslF*, and 12 *CesA* genes were highly expressed at the jointing and maturing stages to promote plant morphogenesis and ensure biomass yield. Therefore, at different developmental stages of leaf and stem, cellulose biosynthesis involved multiple cellulose synthase genes with different expression patterns that cooperated with each other, which may explain, at least partially, why *P. giganteum* is rich in cellulose.

To discover the regulation and expression patterns of genes related to photosynthesis between subgenomes in C4 plants with polyploidization, C4 photosynthesis-related genes in nine gene families were identified in *P. giganteum* (78, A: 39, B:39), which is more than the number in *P. purpureum* (56), *C. americanus* (20), and *S. italica* (24) (Fig. 5c). Most of the C4 photosynthesis-related genes in *P. giganteum* (64/79, 81.01%) were expressed at all seven leaf developmental periods, and 31 homologous 1:1 gene pairs were identified between the A and B subgenomes. The transcriptome analysis showed that there was no significant difference in expression between most of these homologous gene pairs (30/31) across all developmental periods. The C4 photosynthesis-related homologous genes were more highly conserved than other gene families such as protein phosphatases and ubiquitin-mediated proteolysis genes (Fig. 6d). We inferred that these highly conserved C4 photosynthesis-related genes between the A and B subgenomes may have similar expression patterns, which implied that the A and B subgenomes conservatively retained and even expanded photosynthesis-related gene families after the formation of allotetraploid *P. giganteum*. Interestingly, we found that there was at least one A/B homologous gene pair at each step of the C4 photosynthesis pathway. These photosynthesis-related gene pairs were consistently and highly expressed at all developmental stages and may be key candidate genes for genetically improving plant growth and biomass production through the C4 photosynthesis pathway. We analyzed the gene structures of the photosynthesis-related homologous gene pairs, *Pgi0G0022029* and *Pgi0G0048817*, that was significantly differentially expressed, and found that *Pgi0G0048817* had a large deletion that led to the loss of one exon, which may have resulted in its lower expression levels in different developmental periods (Fig. 6e). In summary, the complementarity and additivity of genetic effects of C4 photosynthesis-related genes in the A and B subgenomes may drive massive biomass accumulation and contribute to the rapid growth of *P. giganteum*, which also indicated that both of the A and B subgenomes contributed greatly and almost equally to the highly efficient C4 photosynthesis.

## Discussion

Due to its huge biomass and high application potential as bioenergy and forage grass, *P. giganteum* has been cultivated worldwide. Decoding the genetic base underlying the excellent traits of *P. giganteum* for molecular breeding and genetic improvement is urgently needed to meet the dramatically increased demand for sustainable forage production. Here, we generated a high-quality genome assembly of *P. giganteum*, which represents a major advancement in grass genomics. The quality of the *P. giganteum* assembly (contig N50 88.47 Mb) was significantly higher than other recently published forage genomes including those of *P. purpureum* cv. Purple (contig N50 1.83 Mb) [24], orchard grass (*Dactylis glomerata* L.; contig N50 0.93 Mb) [36], and *Miscanthus lutarioriparius* (contig N50 1.71 Mb) [37], broomcorn millet (contig N50 2.55 Mb) [38], *S. spontaneum Np-X* (contig N50 0.38 Mb) [39], *A. longiglumis* (contig N50 7.30 Mb), *A. insularis* (contig N50 5.64 Mb), *A. sativa* *ssp. *nuda *cv.* Sanfensan (contig N50 75.27 Mb) [40]*.*

Gene family expansion and contraction analysis showed many gene families were significantly expanded in the A and B subgenomes of *P. giganteum* (Fig. 2a), and there was clear functional differentiation of the expanded gene families between the two subgenomes. WGD analysis showed that both subgenomes of *P. giganteum* underwent only the ancient ρ-WGD event shared by grass species, and did not experience any independent WGD event, which is supported by the different types of duplicated genes (Fig. 2d). On the basis of the insertion time of intact LTR-RTs, we detected two LTR peaks at 0.24 Mya and 1.19 Mya in *P. giganteum* (Additional file 2: Figure S31b) and one peak at 1.07 Mya in *C. americanus*. No apparent peak for LTR insertion was detected in *S. italica* or *S. viridis*. These findings indicated that the recent LTR burst in genus *Pennisetum* possibly contributed to their significantly larger genome sizes compared with those in genus *Setaria* (Fig. 3a).

Comparative genomic analysis strongly supported the idea that *P. giganteum* and *P. purpureum* are two different species, despite their high similarity in many morphological features and agronomic traits (Additional file 2: Figure S31; Additional file 1: Table S26). The reconstructed ancestral karyotype of grass species identified massive chromosomal rearrangements among them and a clear change trajectory in chromosome numbers, from ten in *S. bicolor*, nine in *S. viridis*, to seven in *C. americanus* and *P. giganteum*. The synteny analysis showed the A5 segment (50 Mb) in the A5–B14 collinear region was remarkably shorter than the B14 counterpart segment (80 Mb) (Additional file 1: Table S41; Additional file 2: Figure S31c). Interestingly, we found that the length and number of transposable elements in B14 (57.31 Mb) were much more than those in A5 (22.50 Mb). These results indicated that abundant transposable elements were non-uniformly and specifically inserted into chromosome B14, which may provide a potentially excellent resource for studying the mechanism of transposable element amplification or genome evolution.

It has been widely accepted that C4 plants show a distinguishing ability to concentrate CO_2_. Compared with three other closely related C4 plants, *P. giganteum* harbored more copies of C4 photosynthesis-related genes, probably suggesting that *P. giganteum* possesses stronger photosynthesis ability for rapid biomass accumulation. More than 80% of C4 photosynthesis-related genes showed sustained expression across the whole developmental stages of *P. giganteum*, implying that the majority of genes in the C4 photosynthesis pathway were transcriptionally active. Additionally, almost all the homologous gene pairs showed no significant expression bias between two subgenomes, suggestive of the equal contribution of two subgenomes to the photosynthesis. Cellulose is one of the most important components of cell wall in higher plants, which has great impact on the growth and development of plants. On the one hand, more copies of *Ces* superfamily members were identified in *P. giganteum* compared with three other diploid species, implying the contribution of polyploidization to this gene family size. On the other hand, we also observed difference in expression between homologous *Ces* genes in two subgenomes. For example, a pair of *CslF* homologous genes (*Pgi0G0010261* and *Pgi0G0073710*) showed significant difference in expression, and we hypothesized that the difference was possibly caused by the insertion of two LTRs in the genic region of *Pgi0G0073710*. Transcriptome data analysis showed difference in expression between leaf and stem tissues across three developmental stages. In leaf, most of the *CesA* genes (24) showed the highest expression in the maturing stage. By contrast, the numbers of *CesA* genes showing higher transcript abundance were stable across three developmental stages. These results might suggest that the synthesis of cellulose in stem started earlier than in leaf, indicating the distinct roles of different tissues in biomass accumulation.

The existence of subgenome dominance in polyploids has been debated by researchers for a long time and controversies still remain [21, 25, 41–46]. In this study, we compared our results with those in previous studies on allopolyploid species, including *Gossypium hirsutum* [47], *C. purpureus* [24], and *Brassica juncea* [21]. The results showed that the average percentage of homoeologous genes showing differential expression between subgenomes was the lowest in *P. giganteum* (Additional file 2: Figure S32a). To exclude the bias that might be caused by different approaches, we re-analyzed the RNA-seq data from different studies using the same method as used by us. The results indicated that the percentage of homoeologous genes showing biased expression between subgenomes is highest in *P. giganteum* compared with three other polyploid plants (Additional file 2: Figure S32b). However, the ratios of homoeologs that were dominant in two subgenomes were almost equal in *P. giganteum* (Additional file 1: Table S42; Additional file 2: Figure S32b). Therefore, we made a conclusion that no significant subgenome dominance was observed in *P. giganteum* at the overall expression level, which is consistent with several allopolyploid species [21, 48, 49], but a large number of differentially expressed homoeologous genes and homoeolog-specific expressed genes were identified between the two subgenomes in different tissues and at different developmental periods and different developing stages (Fig. 5a, b). The functional enrichment analysis showed that there were significant differences in functional categories of the DEGs between the two subgenomes. The C4 photosynthesis-related genes were expanded in *P. giganteum* and highly conserved between the two subgenomes, which resulted in the balanced and high expression of the homoeologous gene pairs (30/31) between the A and B subgenomes at different developmental periods. This finding indicated that both the A and B subgenomes contributed greatly and almost equally to the highly efficient C4 photosynthesis of *P. giganteum*, which drives massive biomass accumulation and contributes to its rapid growth. Key candidate genes in each gene family in the C4 photosynthesis pathway were identified and were found to be consistently and highly expressed at all developmental stages.

## Conclusions

In summary, this study reported a high-quality genome assembly for the allotetraploid forage grass *P. giganteum*. Our results showed clearly about the functional differentiation between the two subgenomes in *P. giganteum*. The combined comparative genomics analysis and transcriptome meta-analysis showed that complementarity and additivity of genetic effects between the A and B subgenomes may have important impacts on biomass accumulation and rapid growth of *P. giganteum.* The fundamental resources described here will become important references for further studies of the genetic basis of advantageous traits in polyploid species and will facilitate further functional genomics-related research and genetic improvements of *P. giganteum*.

## Methods

### Plant materials, DNA and RNA sample collection and sequencing

*P. giganteum* plants were obtained from National Engineering Research Center of Juncao Technology (Fuzhou City, Fujian Province, China) and planted in Hebei University (Baoding, Hebei Province, China). For genome sequencing, high molecular weight DNA was extracted from the leaves of fresh plants using a DNA extraction kit. Two PCR-free single-molecule real-time (SMRT) bell libraries were constructed from the extracted genomic DNA and sequenced on a third-generation PacBio Sequel II platform (Annoroad Gene Technology, Beijing, China) to obtain high-quality sequencing data. For Hi-C sequencing, the leaves of fresh plants were cross-linked with formaldehyde and the reaction was terminated using glycine solution. The Hi-C library was constructed following the standard protocol and paired-end sequenced on an Illumina HiSeq 2000 platform (Annoroad Gene Technology). For transcriptome sequencing (RNA-seq), *P. giganteum* plants were regenerated by stem propagation and six tissues were harvested from fresh plants, namely parietal tip, inverted third leaf tip, root, stem, leaf, and aboveground and underground parts. The tissue samples were rinsed using ddH_2_O, snap frozen in liquid nitrogen, and stored in − 80 ℃ until used. Three biological replicates were collected for each tissue sample. Total RNA was extracted from the tissue samples using a RNeasy Plant Mini Kit (Qiagen), cDNA libraries were constructed following the manufacturer’s instructions, and paired-end sequencing was performed on a NovaSeq sequencing platform (Illumina) (Berry Genomics, Beijing, China).

### Genome assembly and quality assessment

To estimate the genome size and heterozygosity ratio of *P. giganteum*, k-mer frequency analysis was conducted using the genomic character estimate (GCE v1.0.2) [50] with default parameters. To achieve high-quality genome assembly, high-fidelity (HiFi) reads were generated on a PacBio Sequel II platform using the circular consensus sequencing (CCS) mode. First, the *P. giganteum* genome was de novo assembled using Hifiasm (v0.16.0-r369) [51] with default parameters. Then, the Hi-C reads were used to anchor the assembled contigs onto chromosome pseudomolecules through sorting, orientation, and ordering using 3D-DNA (v170123) [52] and Juicer (v1.6) [53] to yield a final version of the *P. giganteum* genome assembly. The quality of the genome assembly was evaluated using the Benchmarking Universal Single-Copy Orthologs (BUSCO v4.1.4) [54] based on the embryophyta_odb10 dataset, which contains 1614 core genes across land plants and the LTR assembly index [31].

### Genome annotation

Repetitive sequences in the *P. giganteum* genome were annotated using ab initio and homology-based methods. The Tandem Repeat Finder (TRF v4.07b) [55] was used to identify tandem repeat sequences. For the de novo searches, RepeatModeler (v1.0.11), LTR_FINDER (v1.05) [56], LTRharvest (v1.5.11) [57], and LTR_retriever (v1.9) [58] were used to construct de novo repeat libraries, and RepeatMasker (v4.1.1) was used to identify genome-wide repeat sequences based on the de novo library. For homology-based searches, repeat elements were identified by RepeatMasker using a known repeat library (Repbase 15.02) (https://www.girinst.org/server/RepBase/index.php). Additionally, tRNA genes were predicted using tRNAscan-SE [59]. Other non-coding RNAs such as rRNA, miRNA, and snRNA were predicted using INFERNAL (v1.1) [60] by searching against the Rfam database (v9.1) [61].

To predict protein-coding genes in the *P. giganteum* genome, gene models were obtained by combining three prediction approaches, namely ab initio, homology-based, and transcriptome-based methods. For ab initio prediction, PASA (v2.3) [62] was used to predict gene structure using transcripts assembled by Trinity (v2.12) [63]; these transcripts were also used in AUGUSTUS (v3.2.3) [64] to train the gene model. For homology-based prediction, GenomeThreader (v1.7.3) [65] was used to search against the protein sequences of seven plant species, namely *A. thaliana*, *P. purpureum*, *O. sativa*, *S. viridis*, *S. bicolor*, and *C. americanus*. For transcriptome-based prediction, Trinity was used to assemble the RNA-seq data into transcripts, and PASA software was used for gene structure prediction based on transcriptome assembly. In addition, the RNA-seq data were mapped to the *P. giganteum* genome using HISAT2 (v2.2.1) [66], and StringTie (v2.1.6) [67] was used for reference-guided transcript assembly. ORFs (open reading frames) in the assembled transcripts were predicted using TransDecoder (v5.1.0). The prediction results from the different sources were combined using EVidenceModeler (EVM) [68]. The final gene set was evaluated by BUSCO based on the embryophyta_odb10 dataset.

### Subgenome division of the P. giganteum genome

We used three methods to divide the *P. giganteum* genome into subgenomes. First, the next-generation sequencing data of the potential A subgenome donor *C. americanus* was mapped onto the *P. giganteum* genome using BWA-MEM (v0.7.17) [69] and two subgenomes were identified based on mapping rates. Second, protein-coding genes from the two subgenomes of *P. giganteum,S. viridis*, and *C.americanus* were used to construct orthogroups using OrthoFinder [70]. The single-copy gene clusters shared by them were extracted and aligned for each group of homologous chromosomes using MUSCLE (v3.8.1551) [71]. Collinear blocks among the two subgenomes of *P. giganteum*, *C.americanus*, *S. viridis*, and *S. bicolor* were obtained by JCVI (v1.2.1). The 1:1:1:1:1 syntenic blocks (> 50 genes) were selected to construct phylogenetic trees. Third, subgenome-specific repetitive DNA sequences were identified using SubPhaser [72]. Jellyfish v2.2.10 [73] was used to scan for and count 13-bp sequences (13-mers) in the 14 *P. giganteum* chromosomes. After normalizing the differential k-mer matrices, the k-means algorithm was used to cluster chromosomes into two groups (subgenomes).

### Functional annotation

To determine the functional relevance of the predicted gene models in the *P. giganteum* genome, protein-coding genes were retrieved and functionally annotated by BLAST searches against known databases, namely UniProtKB/SwissProt and the NCBI non-redundant (NR) protein database. Gene ontology (GO) functional and Pfam domain annotations were assigned using InterProScan (v5.32) [74, 75]. KEGG orthology (KO) terms for each gene were assigned by homology searches against the hidden Markov model (KOfam) database using KofamScan [76] with default parameters.

### Hi-C read mapping and normalization

Clean Hi-C sequencing data were mapped to *P. giganteum* genome using Bowtie2 with default parameters. The HiC-Pro pipeline (v3.0.0) [77] was used for removing singleton reads, multiple-mapping reads and duplicated read pairs, and only pairs for which both reads are uniquely aligned were kept to identify valid interactions. Raw contact matrices were built using bin sizes of 500 kb and normalized with the iterative correction and eigenvector decomposition (ICE) method using HiC-Pro.

### Identification of A and B compartments

The eigenvector module implemented in the Juicer tools (v1.6) [53] was applied to identify A and B compartments on the 500-kb corrected matrix of each chromosome in *P. giganteum*. For each chromosome, genomic bins for which the first eigenvector (PC1) with a positive or negative value were assigned to compartment A or B, respectively. The genes located in each genomic bin were assigned with the corresponding compartment. To identify the switching of A/B compartment status between two subgenomes, we compared the compartment status change of syntenic gene pairs.

### Variant calling and validation of accuracy rate of single base

The paired-end Illumina reads of *P. giganteum* were aligned to the assembled genome using the BWA-MEM [69] algorithm. Only reads with mapping quality > 30 were retained for downstream analysis. Variant calling was performed using the GATK pipeline (v4.1.6.0; https://github.com/broadinstitute/gatk) with the best practice protocol. After filtering out variants with low coverage or low quality, we selected only the biallelic single-nucleotide polymorphisms (SNPs) for subsequent analysis. The single-base error rate of the assembled genome was calculated as the percentage of homozygous SNP loci in the whole genome, thus the single-base accuracy rate was calculated as: accuracy rate = 1 − error rate.

### Phylogenetic tree construction and evolutionary rate estimation

Orthologous gene clusters in the A and B subgenomes of *P. giganteum* and 11 other representative plants, namely *Setaria viridis* (GenBank: GCA_005286985.1), *Pennisetum purpureum* (https://ngdc.cncb.ac.cn/search/?dbId=gwh &q = GWHAORA00000000 +), *Brachypodium distachyon* (RefSeq: GCF_000005505.3), *Dichanthelium oligosanthes* (GenBank: GCA_001633215.2), *Oryza sativa* (Phytozome), *Cenchrus americanus* (http://cegsb.icrisat.org/ipmgsc/genome.html), *Sorghum bicolor* (RefSeq: GCF_000003195.3), *Setaria italica* (RefSeq: GCF_000263155.2), *Zea mays* (RefSeq: GCF_000005005.2) and two outgroup species, *Arabidopsis thaliana* (GenBank: GCA_000001735.2) and *Glycine max* (GenBank: GCA_000004515.5), were identified using the OrthoFinder (v2.3.14) pipeline [70]. A total of 2561 orthologous groups containing 2961 genes were identified among these species, including 307 single-copy orthologs. These single-copy orthologous genes were used to build a maximum likelihood tree with FastTree (v2.1.9) [78]. The species divergence time was calibrated using r8s [79] based on the TimeTree website [80]. For example, 42.0–52.0 Mya was used for *B. distachyon* and *D. oligosanthes*. FigTree (v1.4.3) was used fto visualize the species tree.

### Gene family analysis

CAFE version 4.0.1 [81] with the default parameters was used to explore the change of gene families. Gene families that were identified in at least five of the species were selected for further analysis. The changes in gene families along each lineage of the phylogenetic tree were evaluated using the built-in model. The change of gene families of all the nodes was analyzed.

### Whole-genome duplication (WGD) and synteny analysis

Synonymous substitution rate (*K*s) estimation was used to detect WGD events in *P. giganteum*. First, the protein sequences of *P. giganteum*, *C. americanus*, *S. bicolor*, and *S. viridis* were aligned against self using BLASTP (E-value = 1 × 10^−10^). Then, the collinear blocks of these plants were identified using MCScanX [82, 83]. The WGD events in each plant species were evaluated based on the *K*s distribution [84]. *K*s values of the homologous gene pairs in the collinear blocks were calculated using the KaKs_calculator (v2.0) [85] with the YN model; the median of *K*s values was considered to be representative of the collinear blocks. The *K*s values of all gene pairs were plotted to identify putative WGD events in *P. giganteum*. The time of WGD and species divergence was estimated as *t* = *K*s/2*r*, where *r* is the neutral substitution rate. In this study, we used a neutral substitution rate of 6.5 × 10^−9^ as reported previously [25].

### Ancestor genome structure inference among P. giganteum and other related grass species

We applied OrthoFinder to find orthogroups among *P. giganteum, C. americanus, S. viridis, Z. mays*, and *S. bicolor*. The orthogroups were filtered to generate an input file for DRIMM-Synteny [86] following the instructions in the processDrimm pipeline (https://github.com/xjtu-omics/processDrimm). Then, DRIMM-Synteny was run to generate synteny and blocks information. The DRIMM-Synteny output files were processed to retain the synteny blocks that met the expected copy number as the input file for the IAGS pipeline [87] (https://github.com/xjtu-omics/IAGS) using the Python script processDrimm.py (https://github.com/xjtu-omics/processDrimm/processDrimm.py). Finally, the ancestor genome structure was inferred following the IAGS pipeline instructions.

### Genome-wide differential expression analysis

Syntenic gene pairs between the A and B subgenomes of *P. giganteum* were obtained using MCScanX with the default parameters, and 21,258 reciprocal best-match gene pairs that met the 1:1 relationship between the two subgenomes were identified as allelic gene pairs. To investigate differences in gene expression between the two subgenomes of *P. giganteum*, we calculated the FPKM (fragments per kilobase of exon model per million mapped fragments) values of the allelic genes in six tissues, namely apical tip, inverted third tip, root, stem, and the aboveground and underground parts of seedling, and at seven developmental stages of leaf and stem. Differentially expressed genes were identified using the DESeq2 (v1.32.0) package [88] with a minimum of twofold differential expression (|log2Foldchange|> 1) and *P*_adj_ < 0.05. Sequence alignment and phylogenetic tree construction of cellulose synthase and C4 photosynthesis-related genes were performed using MUSCLE (v3.8.1551) [71] and IQ-TREE 2 [89]. The phylogenetic tree was visualized using iTOL v5 [90].

### Comparative genomic analysis of P. giganteum and P. purpureum

To compare the similarity of amino acid sequences between *P. giganteum* and *P. purpureum*, BLASTP search was performed with the E-value cutoff = 1 × 10^−3^, and the best hit was used for sequence similarity analysis. Additionally, OrthoFinder [70] was used to assign orthogroups (OGs) to the subgenomes of *P. giganteum* and *P. purpureum.* Genome-wide synteny analysis between *P. giganteum* and *P. purpureum* was conducted using JCVI with the default parameters. MUMmer (v4.0.0beta2) [91] was employed for whole genome alignment between *P. giganteum* and *P. purpureum*, and SyRI [92] was used to identify genomic structural variation (SVs) between them based on whole genome alignment result. The visualization of genome syntenic and rearrangement regions was performed using plotsr [93]. The original SVs generated by SyRI were filtered to retain only large SVs (> 50 bp) with the scripts SyRI_Parse.pl and Covert_CPV.pl in the pipeline GenomeSV (https://github.com/wolongac/GenomeSV). To compare the difference in nucleotide substitution rate between two species, *K*a/*K*s ratios were calculated for each subgenome of two species using KaKs_Calculator 2.0 [85], respectively.

## Supplementary Information

**Additional file 1: Table S1. **Summary of DNA sequencing data for *Pennisetum giganteum*. **Table S2.** Genome size estimation result of *P. giganteum* based on k-mer frequency analysis. **Table S3.** Statistics of the *P. giganteum* genome assembly. **Table S4.** The number of contigs and gaps in chromosomes of *P. giganteum*. **Table S5.** Statistics of chromosome-anchored contigs in subgenomes of *P. giganteum*. **Table S6.** BUSCO analysis of *P. giganteum* genome assembly. **Table S7.** The mapping rate of RNA-seq data to the *P. giganteum* genome. **Table S8.** Summary of homozygous SNPs detected in genome resequencing data of *P. giganteum*. **Table S9.** Statistics of repeat contents in the A and B subgenomes of *P. giganteum*. **Table S10.** Overview of protein-coding genes and transposable elements on chromosomes of A and B subgenomes. **Table S11.** Summary of intact LTR-RTs identified in the *P. giganteum* genome. **Table S12.** Statistics of gene annotation in *P. giganteum*. **Table S13.** Functional annotation of the predicted *P. giganteum* genes. **Table S14.** Mapping rates of pearl millet short reads on chromosomes of *P. giganteum*. **Table S15. **Summary of subgenome-specifically enriched 13-mers in *P. giganteum*. **Table S16.** Orthologous groups identified in *P. giganteum *and other plant species. **Table S17.** Distribution of different categories of gene families in *P. giganteum* and other plant species. **Table S18.** GO enrichment analysis of expanded genes of subgenome A and B in *P. giganteum*. **Table S19.** KEGG enrichment analysis of expanded genes of subgenome A and B in *P. giganteum*. **Table S20. **GO enrichment analysis of conserved genes of subgenome A in *P. giganteum*. **Table S21. **GO enrichment analysis of conserved genes of subgenome B in *P. giganteum*. **Table S22.** GO enrichment analysis of unique genes *P. giganteum* A and B compared with *C. americanus*. **Table S23.** GO enrichment analysis of unique genes in *P. giganteum *A and B compared with *S. viridis*. **Table S24.** GO enrichment analysis of unique genes in *P. giganteum *A and B compared with *S. bicolor*. **Table S25.** GO enrichment analysis of unique genes in *P. giganteum* A and B. **Table S26.** Statistics of number and length of SVs between different plant species. **Table S27.** Summary of highly conversed genes of A and B subgenome in *P. giganteum* relative to *C. americanus* and *S. viridis*. **Table S28.** Summary of the ratio of 3D chromatin A/B compartments between the syntenic gene pairs of A and B subgenomes. **Table S29.** Comparison of gene and exon in different tissues of *P. giganteum*. **Table S30.** The number of highly expressed genes in subgenomes of *P. giganteum*. **Table S31.** Summary of differentially expressed genes in inter-tissue comparison groups. **Table S32.** Summary of expressed homoeologous genes in different tissues of the AB subgenome. **Table S33.** Summary of homoeolog-specific genes in different tissues of the AB subgenome. **Table S34.** Summary of cellulose synthase related gene families in *P. giganteum* and *S. viridis*. **Table S35.** Summary of expression levels of cellulose synthesis related genes in different tissues of *P. giganteum*. **Table S36.** Distribution of cellulose synthase genes on chromosomes of *P. giganteum*. **Table S37.** Comparison of expression levels of homoeologous gene pairs between the A and B subgenomes in *P. giganteum*. **Table S38.** Summary of differentially expressed genes in seven stages of *P. giganteum*. **Table S39.** GO enrichment analysis of DEGs in leaves at seven stages in *P. giganteum*. **Table S40.** GO enrichment analysis of DEG in stems at seven stages in *P. giganteum.***Table S41.** Statistics of all and full-length LTRs in A5-B14 syntenic regions. **Table S42.** Homoeolog expression dominance in *P. giganteum* and three other polyploid species.

**Additional file 2: Figure S1.** Interaction frequency distribution of Hi-C links among chromosomes. The Hi-C version of *Pennisetum giganteum* genome assembly was generated by 3D-DNA pipeline. **Figure S2.** K-mer frequency distribution. The sharp peak depthwas 40 in the distribution. **Figure S3.** Mapping rates of *C. americanus* short reads on chromosomes of *P. giganteum*. **Figure S4. **Unsupervised hierarchical clustering of differential 15-mer sequences for each chromosome in *P. giganteum*. The clustering result confirmed that the genome is successfully phased into two subgenomes based on distinct patterns of differential k-mers. **Figure S5.** Phylogenetic trees of homologous chromosomes among the two subgenomes of *P. giganteum*, *C. americanus*, and *S. viridis* based on single-copy orthologs. All phylogenetic trees displayed a similar topology and validated that subgenome A of *P. giganteum* showed a closer phylogenetic relationship with *C. americanus* than did subgenome B. **Figure S6.** The syntenic blocks across two subgenomes of *P. giganteum*, *C. americanus*, *S. viridis* and *S. bicolor*. The circled numbers below the ideograms marked the big syntenic blocksthat were selected for constructing phylogenetic trees based on syntenic gene pairs. **Figure S7. **Phylogenetic analysis based on homologous genes within syntenic blocks across two subgenomes of *P. giganteum*, *C. americanus*, *S. viridis* and *S. bicolor*. The chromosome numberings refer to the block division of Figure S6. **Figure S8.** Bar plot showing the numbers of genes encoding proteins containing the Pfam domain PF00195 that are involved in flavonoid biosynthesis with detectable expression in the six tissues of *P. giganteum*. P-values were calculated using the Fisher’s exact test. **Figure S9.** GO and KEGG enrichment analysis of conserved genes between subgenomes A and B. GO enrichment analysis of conserved genes in subgenome A. GO enrichment analysis of conserved genes in subgenome B. KEGG enrichment analysis of conserved genes in subgenome A. KEGG enrichment analysis of conserved genes in subgenome B. **Figure S10.** GO enrichment analysis of unique genes between *P. giganteum* A, B and three related species. GO enrichment analysis of unique genes in *P. giganteum* A. GO enrichment analysis of unique genes in *P. giganteum* B. GO enrichment analysis of unique genes in *P. giganteum* A. GO enrichment analysis of unique genes in *P. giganteum* B. GO enrichment analysis of unique genes in *P. giganteum* A. GO enrichment analysis of unique genes in *P. giganteum* B. **Figure S11.** GO enrichment analysis of unique genes between *P. giganteum* A and B. GO enrichment analysis of unique genes in *P. giganteum* A. GO enrichment analysis of unique genes in *P. giganteum* B. **Figure S12.** Boxplot showing the average expression of three classes of genes in two subgenomes of *P. giganteum*, including conserved genes, unique genes and other genes. **, significant; ****, extremely significant; ns, not significant. **Figure S13.** Classification of gene duplicates in the A and B subgenomes of *P. giganteum* and *P. purpureum*. The origins of duplicated genes were categorized into five types: WGD/segmental duplication, tandem duplication, proximal duplication, dispersed duplication, and singleton. P-values were calculated using the Fisher’s exact test. **Figure S14.** Genome-wide syntenic analysis between *C. americanus* and two subgenomes of *P. giganteum*. Synteny between *P. giganteum* A and *C. americanus*. Synteny between *P. giganteum* B and *C. americanus*. Pairwise whole-genome alignments across the A and B subgenomes of *P. giganteum* and *C. americanus*. **Figure S15.** Comparison of conserved gene numbers between two subgenomes of *P. giganteum* and *C. americanus*, *S. viridis*, respectively. **Figure S16.** Chromosome rearrangements and validation by Hi-C interaction evidence. The verification of inverted fragments involved in chromosome rearrangement was performed using Hi-C heatmap between two subgenomes of *P. giganteum*. Collinearity and chromosome rearrangements between the A and B subgenomes of *P. giganteum*. **Figure S17. **Inference of centromere positions based on the distribution of three kinds of genomic features along the 14 *P. giganteum* chromosomes. Orange curves indicate the density of long terminal repeat retrotransposonson each chromosome of *P. giganteum* with a window size of 1 Mb. Green curves indicate the distribution protein-coding genes on each chromosome of *P. giganteum*. Blue curves indicate the distribution of the most abundant 20-k-mers along each chromosome. Red dashed lines corresponds to the putative centromeric region of each *P. giganteum* chromosome. **Figure S18.** Distribution of A/B compartment status conservation or switching in *P. giganteum*. Stacked bar chart showing the distribution of different types of chromatin A/B compartment status for homologous chromosome pairs in *P. giganteum*. A-to-A and B-to-B represent conserved chromatin compartment status between syntenic gene pairs, while A-to-B and B-to-A denote the switching of chromatin compartment status between syntenic gene pairs. Comparison of A/B compartment status switching between non-rearranged and rearranged genomic regions. Statistical significance was determined using one-tailed Fisher’s exact test. **Figure S19.** Preliminary analysis of six tissues for differential expression analysis in *P. giganteum*. Correlation analysis for biological replicates of six tissues in *P. giganteum*. PCA clustering of all RNA-seq samples in *P. giganteum*. **Figure S20.** Preliminary analysis of six tissues for differential expression analysis of subgenome A and B in *P. giganteum*. Distribution of normalized expression levels in six tissues of *P. giganteum*. Correlation analysis for biological replicates of six tissues in *P. giganteum*. PCA clustering of all RNA-seq samples in *P. giganteum* A and B . **Figure S21.** GO enrichment of differentially expressed genesbetween paired tissues in *P. giganteum*. Enriched biological processes for DEGs between paired tissues in *P. giganteum*. Enriched molecular functions for DEGs between paired tissues in *P. giganteum*. Enriched cellular components for DEGs between paired tissues in *P. giganteum*. **Figure S22.** Identification of DEGs and GO enrichment analysis. Upset plot showing DEGs among six tissues in the A subgenome of *P. giganteum*. Upset plot showing DEGs among six tissues in the B subgenome of *P. giganteum*. GO enrichment of DEGs of six tissues in the A and B subgenomes of *P. giganteum*. **Figure S23.** Identification of homoeologous differentially expressed genesand clustering analysis. Upset plot of HDEGs among six tissues of the A subgenome in *P. giganteum*. Upset plot of HDEGs among six species of the B subgenome in *P. giganteum*. Heatmap showing the expression pattern of consistent group of homoeolog-specific expressed genesof six tissues in the A and B subgenomes of *P. giganteum*. **Figure S24.** Comparison of gene level characteristics between the A and B subgenomes of *P. giganteum*. **Figure S25. **Preliminary analysis of seven stages for differential expression analysis in *P. giganteum*. Correlation analysis for biological replicates of leaf samples at seven stages in *P. giganteum*. PCA clustering of all leaf RNA-seq samples in *P. giganteum*. **Figure S26.** Expression patterns of differentially expressed genes in leaves at different growth stages. The heatmap shows the log2-based FPKM+0.01 at seven growth stages. Clustering heatmap of differentially expressed genes in leaves at different growth stages. **Figure S27.** Preliminary analysis of stem RNA-seq samples at seven stages for differential expression analysis in *P. giganteum*. Correlation analysis for biological replicates of stem samples at seven stages in *P. giganteum*. PCA clustering of all stem RNA-seq samples in *P. giganteum*. **Figure S28.** Clustering heatmap of differentially expressed genes in stems at different growth stages. Expression of differentially expressed genes in stems at different growth stages. The heatmap shows the log2-based FPKM+0.01 at seven growth stages. **Figure S29.** Expression of cellulose synthase-related genes in leaves at different growth stages. The heatmap shows the log2-based FPKM+0.01 at seven growth stages. **Figure S30. **Expression of cellulose synthase-related genes in stems at different growth stages. The heatmap shows the log2-based FPKM+0.01 at seven growth stages. Gene expression heatmap for cellulose synthase genes that were expressed at least one time point in stems. **Figure S31.** Distribution of intact LTRs in *P. giganteum* and comparison of LTRs in the syntenic region of chromosomes A5 and B14. Boxplot showing the distribution of LTR Assembly Indexscores on each chromosome of *P. giganteum*. The gray dotted line represents the average LAI score across the whole genome. The density plot of insertion time of *Gypsy *and *Copia *retrotransposons in *C. americanus* and subgenomes A and B of *P. giganteum*. The *Gypsy*-type LTR peak positions of the three species were very close, while the *Copia*-type LTR peak positions differed greatly between *C. americanus* and *P. giganteum*, thus resulting in the two peaks of LTR insertion in *P. giganteum*. The distribution of repeat sequences in syntenic regions of A5and B14. Intact LTRs are shown as green blocks and genes are indicated as blue blocks. The distribution of repeat sequences in a microsynteny block of A5and B14. Intact LTRs are shown as red blocks and other types of TEs are indicated as blue blocks. **Figure S32. **Comparison of differentially expressed genesand balanced genes between subgenomes across different polyploid species. Distribution of DEGs and balanced genes between subgenomes in different studies. Distribution of DEGs and balanced genes between subgenomes in different studies using the same method as used by us.

## Data Availability

All data generated or analyzed during this study are included in this published article, its supplementary information files and publicly available repositories. PacBio HiFi whole-genome sequencing reads, Illumina genomic (including Hi-C experiment) and transcriptomic reads generated in this study have been deposited in the Genome Sequence Archive [94] in National Genomics Data Center [95], China National Center for Bioinformation / Beijing Institute of Genomics, Chinese Academy of Sciences (GSA: PRJCA011649) and are publicly accessible at https://ngdc.cncb.ac.cn/gsa. Additionally, the genome assembly and annotation data have been deposited in the Figshare database (https://doi.org/10.6084/m9.figshare.23118794) [96].

## Abbreviations

CCS: Circular consensus sequencing
Hi-C: High-throughput chromosome conformation capture
BUSCO: Benchmarking Universal Single-Copy Orthologs
RNA-seq: RNA sequencing
LTR-RTs: Long terminal repeat retrotransposons
Mya: Million years ago
WGD: Whole-genome duplication
GO: Gene ontology
KEGG: Kyoto Encyclopedia of Genes and Genomes
KO: KEGG orthology
BH: Benjamini-Hochberg
CHS/STS: Chalcone and stilbene synthase
OGs: Orthogroups
SV: Structural variation
HDEG: Homoeologous differentially expressed gene
HSEG: Homoeolog-specific expressed gene
*CesA*: Cellulose synthase
*Csl*: Cellulose synthase-like
DEG: Differentially expressed gene
SMRT: Single-molecule real-time
HiFi: High-fidelity
EVM: EVidenceModeler
SNP: Single-nucleotide polymorphism
*K*s: Synonymous substitution rate
*K*a: Nonsynonymous substitution rate
FPKM: Fragments per kilobase of exon model per million mapped fragments
NR: Non-redundant
