# Supplementary material for: Differential subgenome expression underlies biomass accumulation in allotetraploid Pennisetum giganteum
Source: BMC Biol. 2023 Jul 21;21:161. doi: 10.1186/s12915-023-01643-w (PMC10362693; doi:10.1186/s12915-023-01643-w)
Supplement: Supplementary file 2 — Additional file 2: Figure S1. Interaction frequency distribution of Hi-C links among chromosomes. The Hi-C version of Pennisetum giganteum genome assembly was generated by 3D-DNA pipeline. Figure S2. K-mer frequency distribution. The sharp peak depthwas 40 in the distribution. Figure S3. Mapping rates of C. americanus short reads on chromosomes of P. giganteum. Figure S4. Unsupervised hierarchical clustering of differential 15-mer sequences for each chromosome in P. giganteum. The clustering result confirmed that the genome is successfully phased into two subgenomes based on distinct patterns of differential k-mers. Figure S5. Phylogenetic trees of homologous chromosomes among the two subgenomes of P. giganteum, C. americanus, and S. viridis based on single-copy orthologs. All phylogenetic trees displayed a similar topology and validated that subgenome A of P. giganteum showed a closer phylogenetic relationship with C. americanus than did subgenome B. Figure S6. The syntenic blocks across two subgenomes of P. giganteum, C. americanus, S. viridis and S. bicolor. The circled numbers below the ideograms marked the big syntenic blocksthat were selected for constructing phylogenetic trees based on syntenic gene pairs. Figure S7. Phylogenetic analysis based on homologous genes within syntenic blocks across two subgenomes of P. giganteum, C. americanus, S. viridis and S. bicolor. The chromosome numberings refer to the block division of Figure S6. Figure S8. Bar plot showing the numbers of genes encoding proteins containing the Pfam domain PF00195 that are involved in flavonoid biosynthesis with detectable expression in the six tissues of P. giganteum. P-values were calculated using the Fisher’s exact test. Figure S9. GO and KEGG enrichment analysis of conserved genes between subgenomes A and B. GO enrichment analysis of conserved genes in subgenome A. GO enrichment analysis of conserved genes in subgenome B. KEGG enrichment analysis of conserved genes in subgenome A. KEGG [file 12915_2023_1643_MOESM2_ESM.pdf]

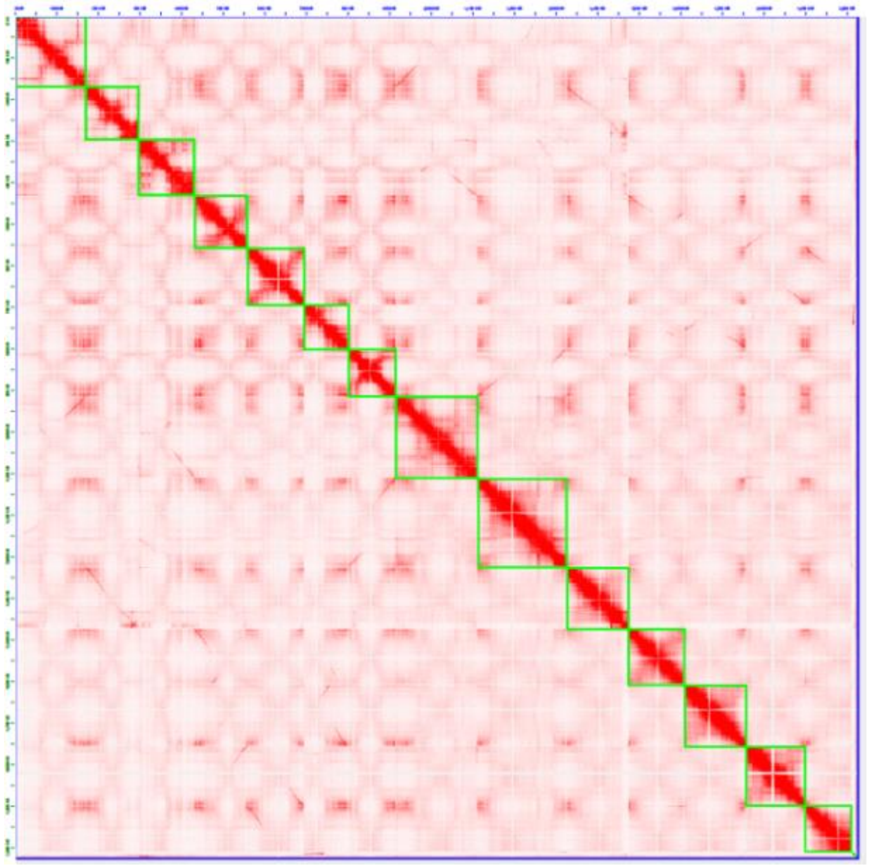

**Figure S1.** Interaction frequency distribution of Hi-C links among chromosomes. The Hi-C version of *Pennisetum giganteum* genome assembly was generated by 3D-DNA pipeline.

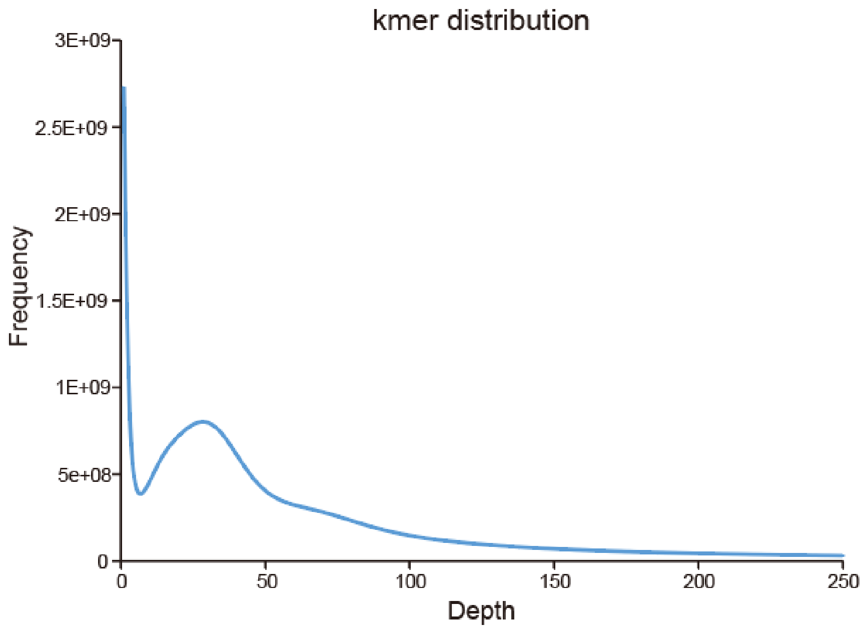


**Figure S2.** K-mer frequency distribution. The sharp peak depth (μ) was 40 in the distribution.

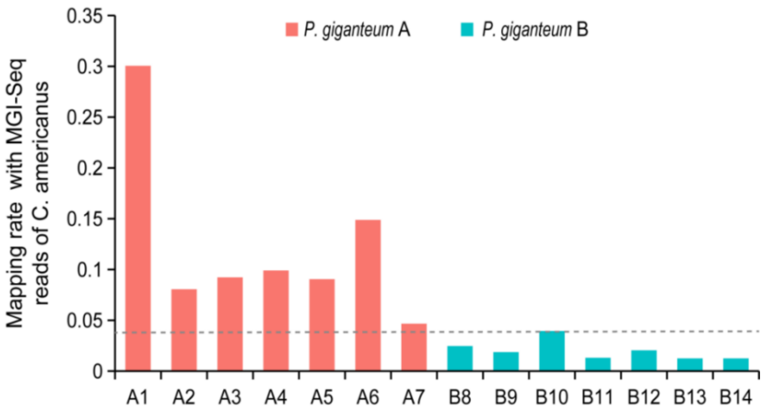


**Figure S3.** Mapping rates of *C. americanus* short reads on chromosomes of *P. giganteum*.

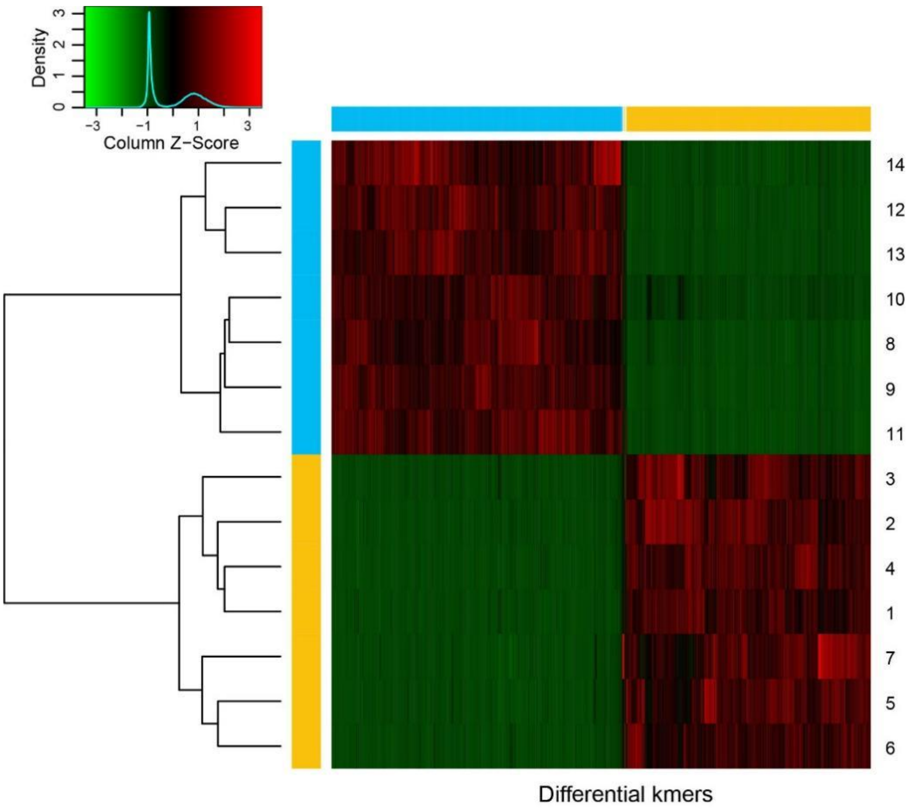


**Figure S4.** Unsupervised hierarchical clustering of differential 15-mer sequences for each chromosome in *P. giganteum*. The clustering result confirmed that the genome is successfully phased into two subgenomes based on distinct patterns of differential k-mers.

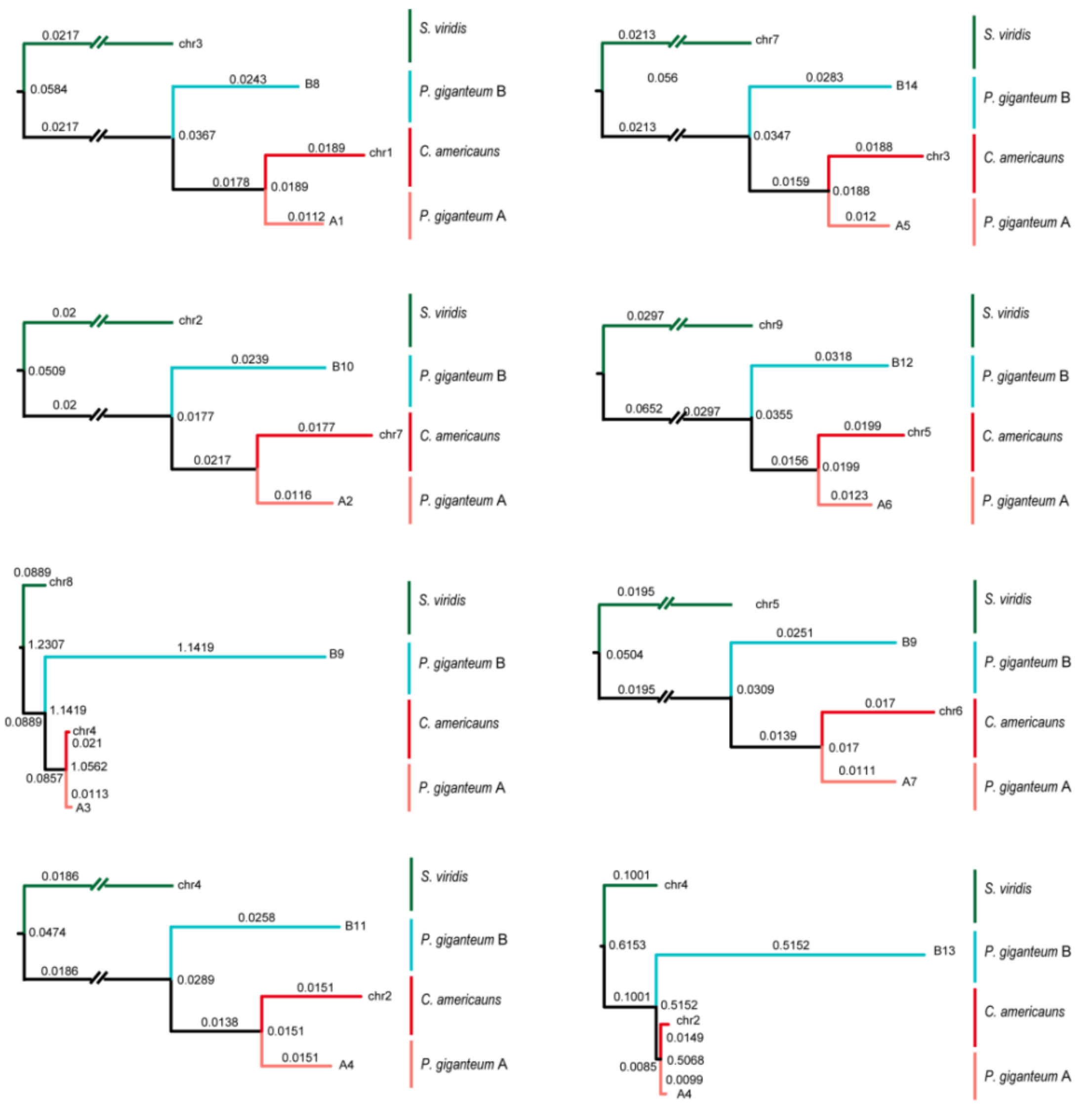


**Figure S5.** Phylogenetic trees of homologous chromosomes among the two subgenomes of *P. giganteum*, *C. americanus*, and *S. viridis* based on single-copy orthologs. All phylogenetic trees displayed a similar topology and validated that subgenome A of *P. giganteum* showed a closer phylogenetic relationship with *C. americanus* than did subgenome B.

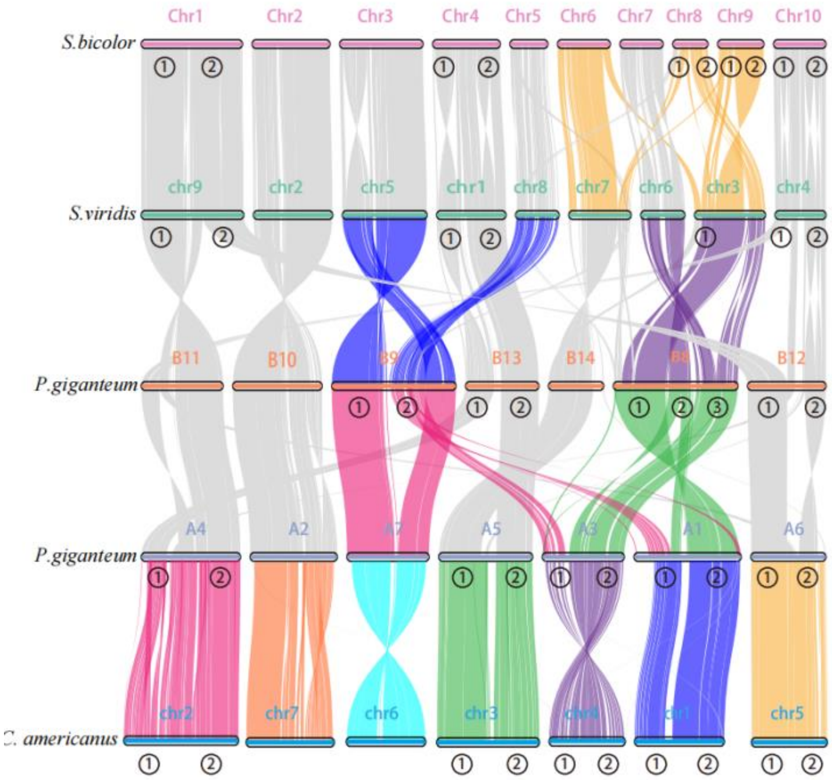


**Figure S6.** The syntenic blocks across two subgenomes of *P. giganteum*, *C. americanus*, *S. viridis* and *S. bicolor*. The circled numbers below the ideograms marked the big syntenic blocks (>30 gene pairs) that were selected for constructing phylogenetic trees based on syntenic gene pairs.

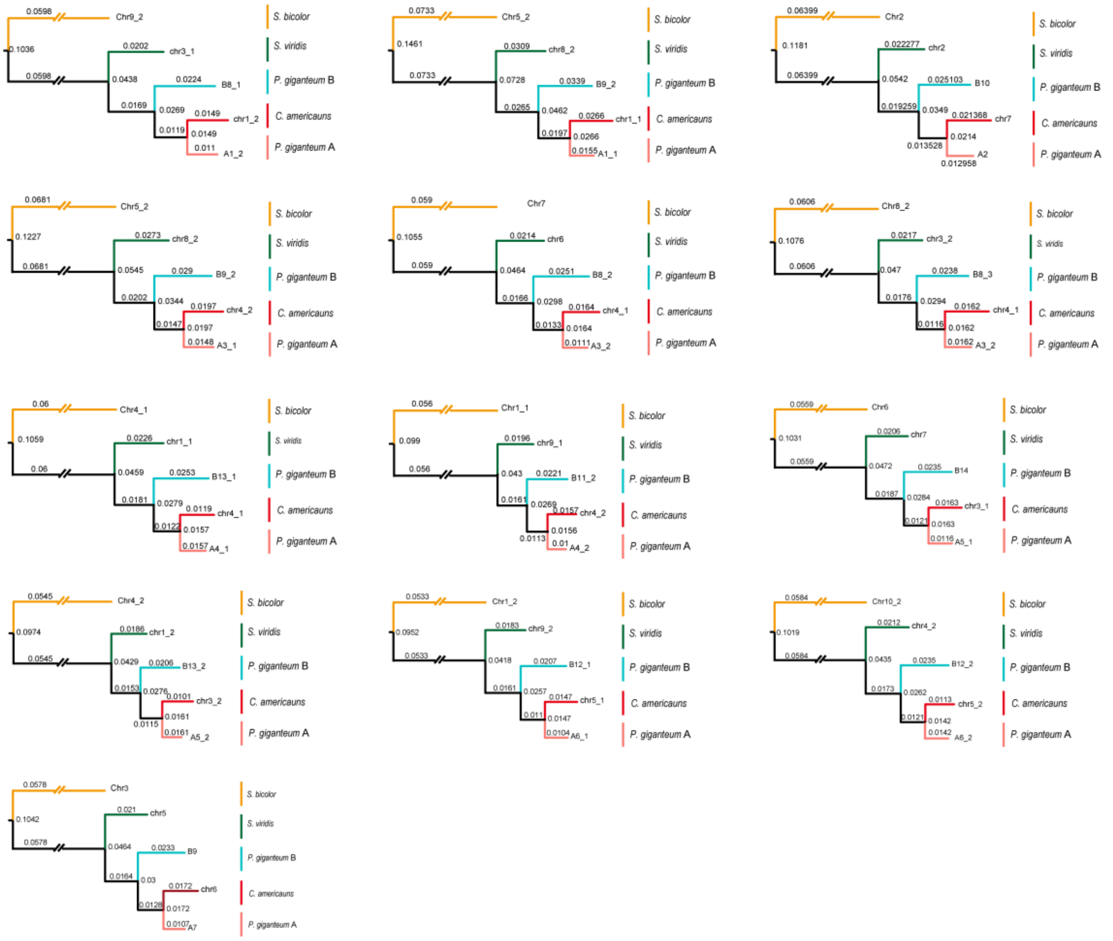


**Figure S7.** Phylogenetic analysis based on homologous genes within syntenic blocks across two subgenomes of *P. giganteum*, *C. americanus*, *S. viridis* and *S. bicolor*. The chromosome numberings refer to the block division of Figure S6.

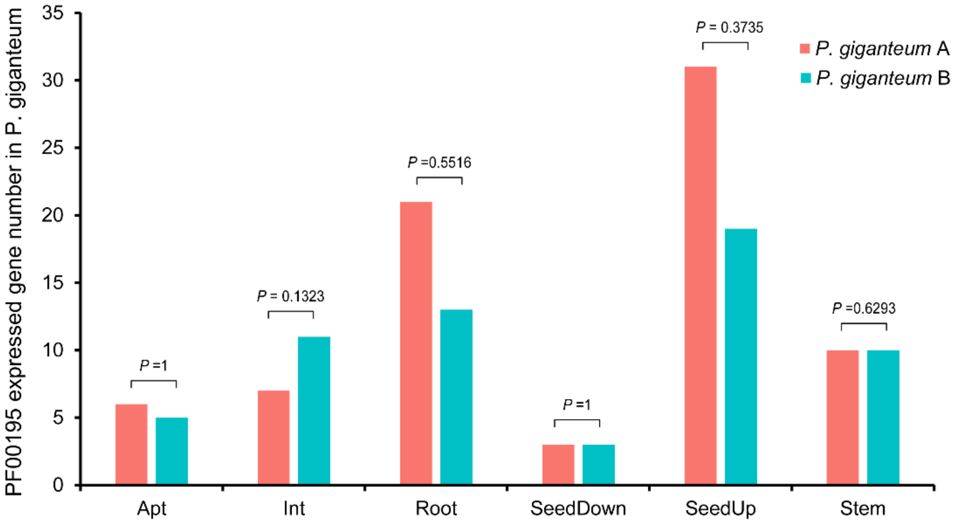


**Figure S8.** Bar plot showing the numbers of genes encoding proteins containing the Pfam domain PF00195 that are involved in flavonoid biosynthesis with detectable expression in the six tissues of *P. giganteum*. P-values were calculated using the Fisher's exact test.

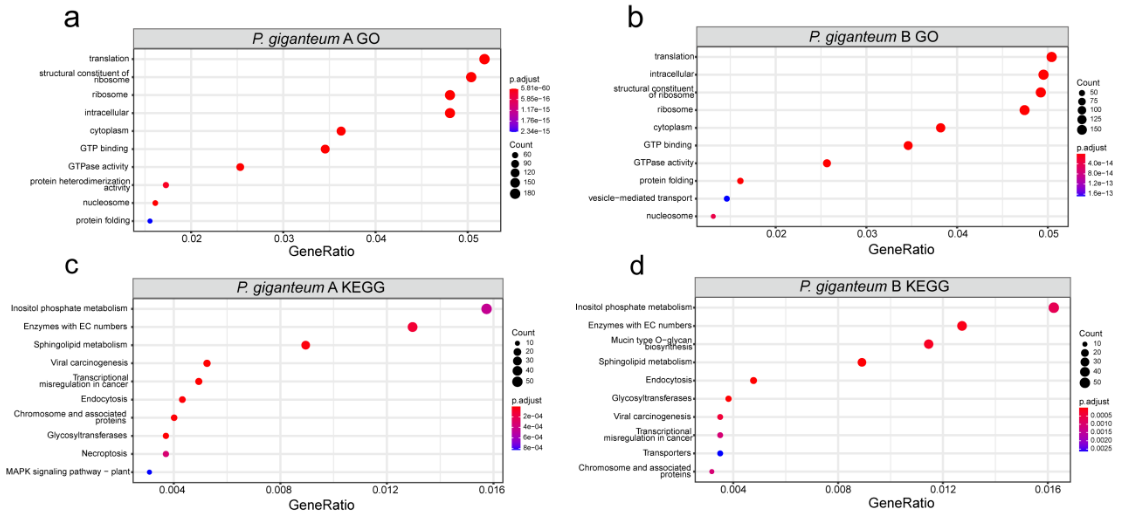


**Figure S9.** GO and KEGG enrichment analysis of conserved genes of sub-genome A and B. **(a)** GO enrichment analysis of conserved genes in subgenome A. **(b)** GO enrichment analysis of conserved genes in subgenome B. **(c)** KEGG enrichment analysis of conserved genes in subgenome A. **(d)** KEGG enrichment analysis of conserved genes in subgenome B.

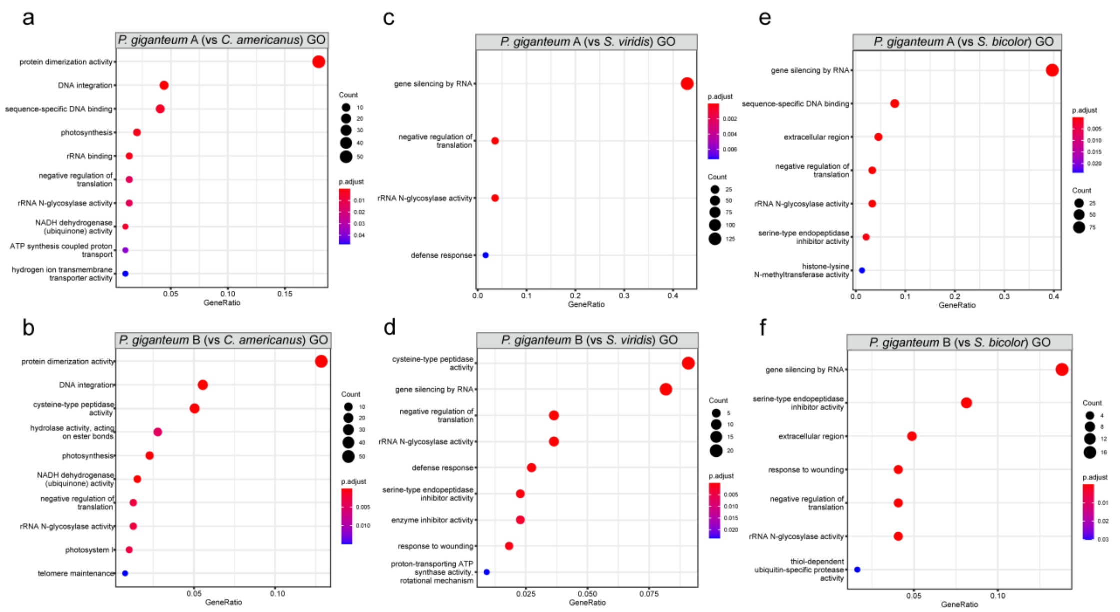


**Figure S10.** GO enrichment analysis of unique genes between *P. giganteum* A, B and three related species. **(a)** GO enrichment analysis of unique genes in *P. giganteum* A (vs *C. americanus*). **(b)** GO enrichment analysis of unique genes in *P. giganteum* B (vs *C. americanus*). **(c)** GO enrichment analysis of unique genes in *P. giganteum* A (vs *S. viridis*). **(d)** GO enrichment analysis of unique genes in *P. giganteum* B (vs *S. viridis*). **(e)** GO enrichment analysis of unique genes in *P. giganteum* A (vs *S. bicolor*). **(f)** GO enrichment analysis of unique genes in *P. giganteum* B (vs *S. bicolor*).

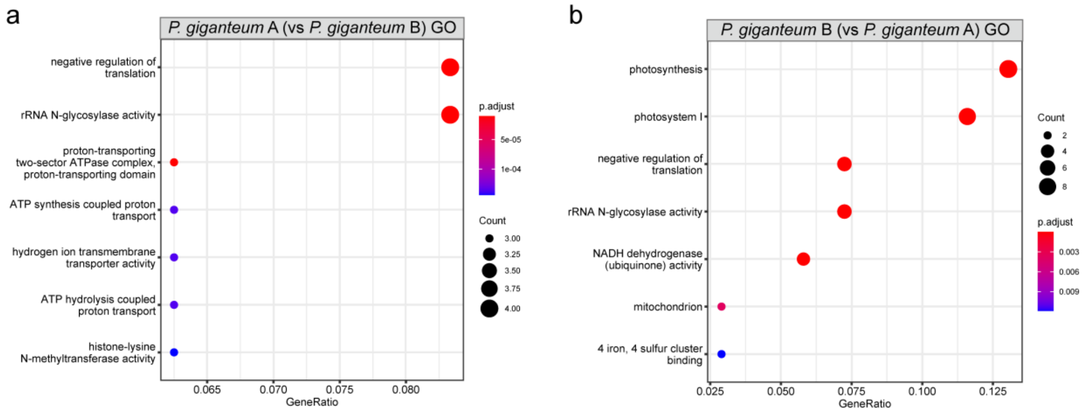


**Figure S11.** GO enrichment analysis of unique genes between *P. giganteum* A and B. **(a)** GO enrichment analysis of unique genes in *P. giganteum* A (vs *P. giganteum* B). **(b)** GO enrichment analysis of unique genes in *P. giganteum* B (vs *P. giganteum* A).

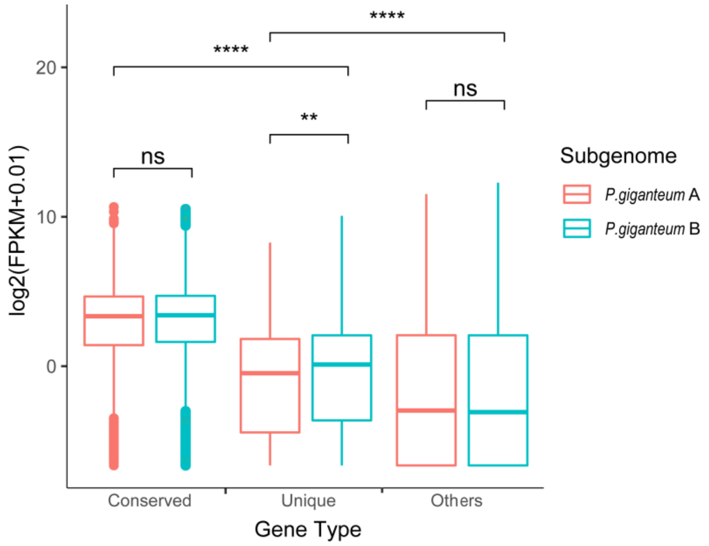


**Figure S12.** Boxplot showing the average expression of three classes of genes in two subgenomes of *P. giganteum*, including conserved genes, unique genes and other genes. **, significant; ****, extremely significant; ns, not significant.

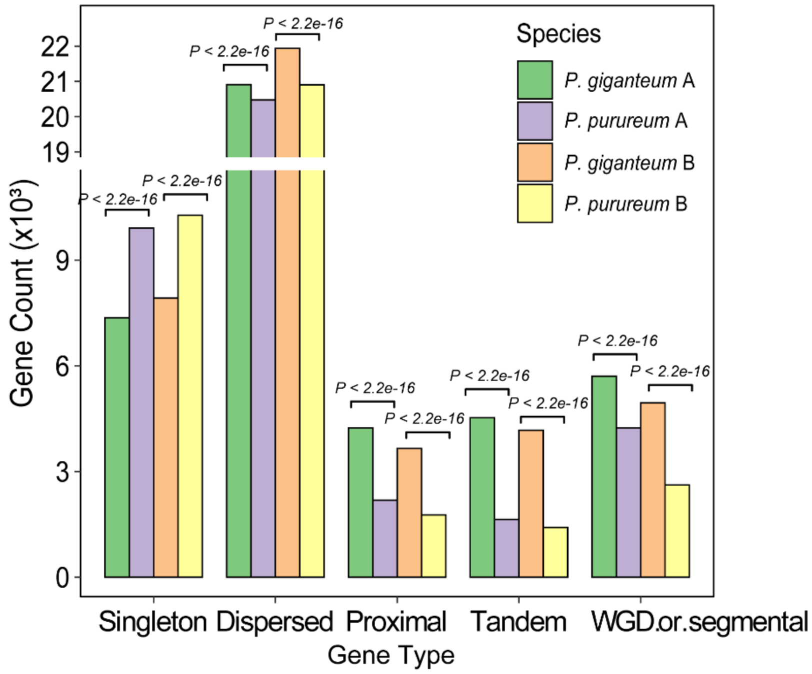


**Figure S13.** Classification of gene duplicates in the A and B subgenomes of *P. giganteum* and *P. purpureum*. The origins of duplicated genes were categorized into five types: WGD/segmental duplication, tandem duplication, proximal duplication, dispersed duplication, and singleton. P-values were calculated using the Fisher's exact test.

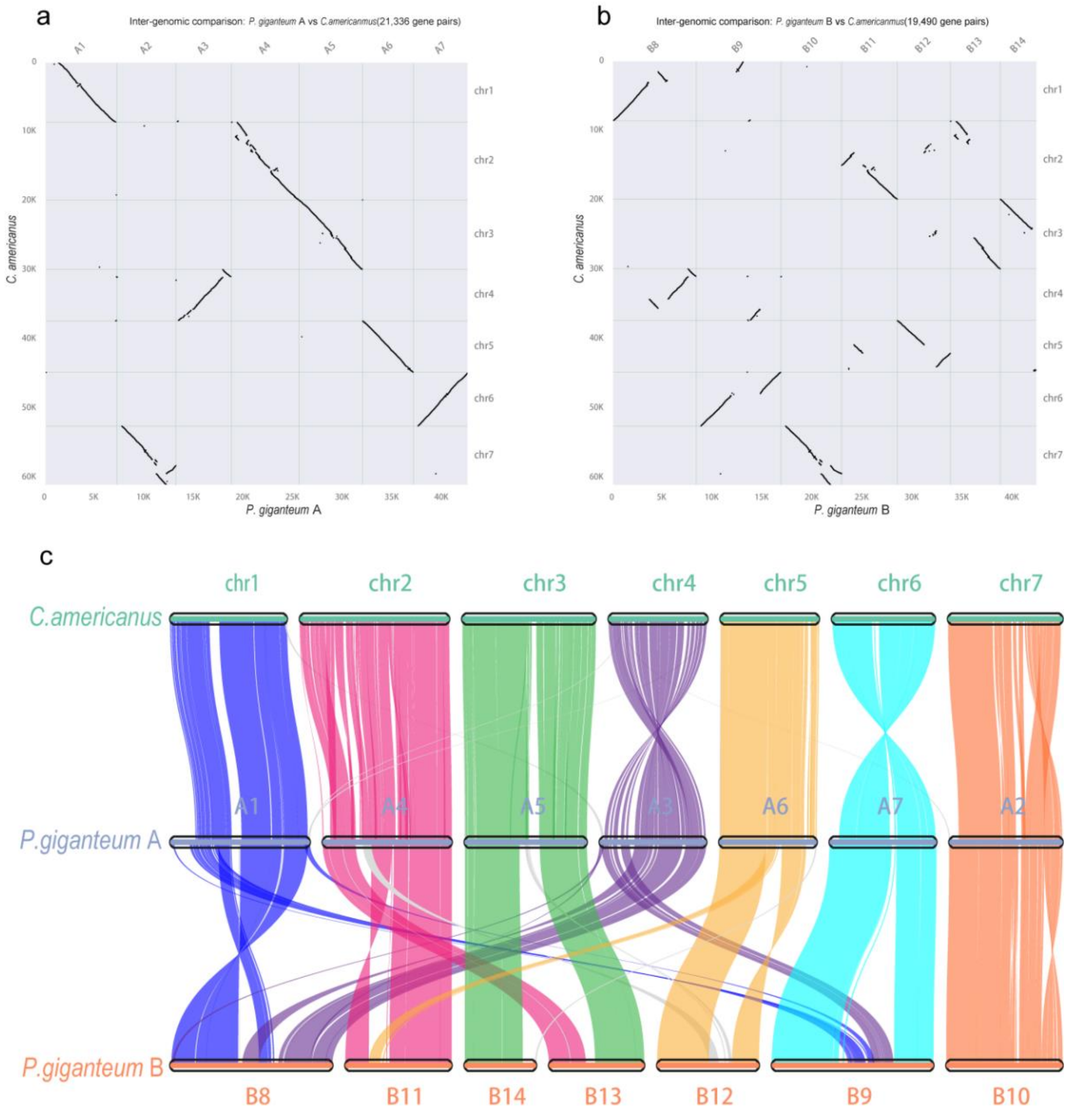


**Figure S14.** Genome-wide syntenic analysis between *C. americanus* and two subgenomes of *P. giganteum*. **(a)** Synteny between *P. giganteum* A and *C. americanus.* **(b)** Synteny between *P. giganteum* B and *C. americanus.* **(c)** Pairwise whole-genome alignments across the A and B subgenomes of *P. giganteum* and *C. americanus*.

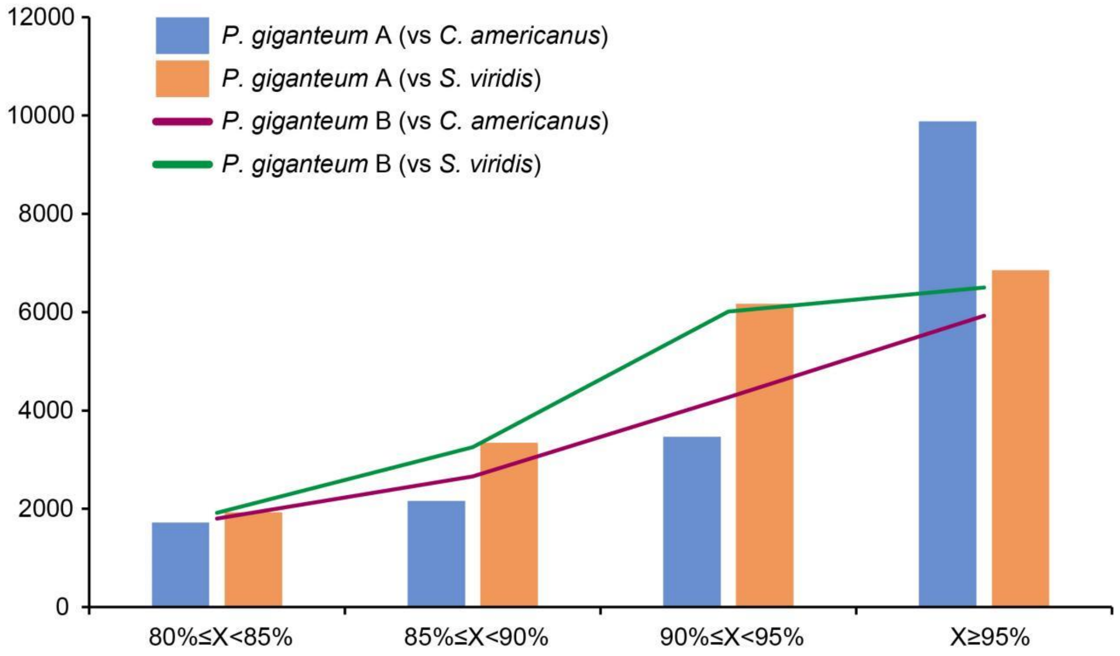


**Figure S15.** Comparison of conserved gene numbers between two subgenomes of *P. giganteum* and *C. americanus*, *S. viridis*, respectively.

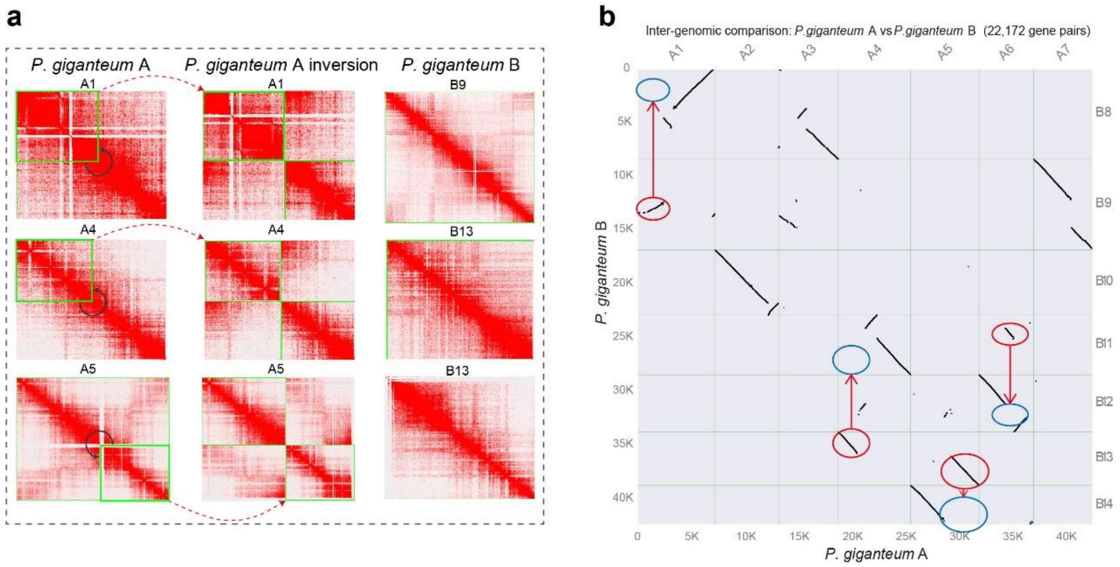


**Figure S16.** Chromosome rearrangements and validation by HiC interaction evidence. **(a)** The verification of inverted fragments involved in chromosome rearrangement was performed using HiC heatmap between two subgenomes of *P. giganteum.* **(b)** Collinearity and chromosome rearrangements between the A and B subgenomes of *P. giganteum.*

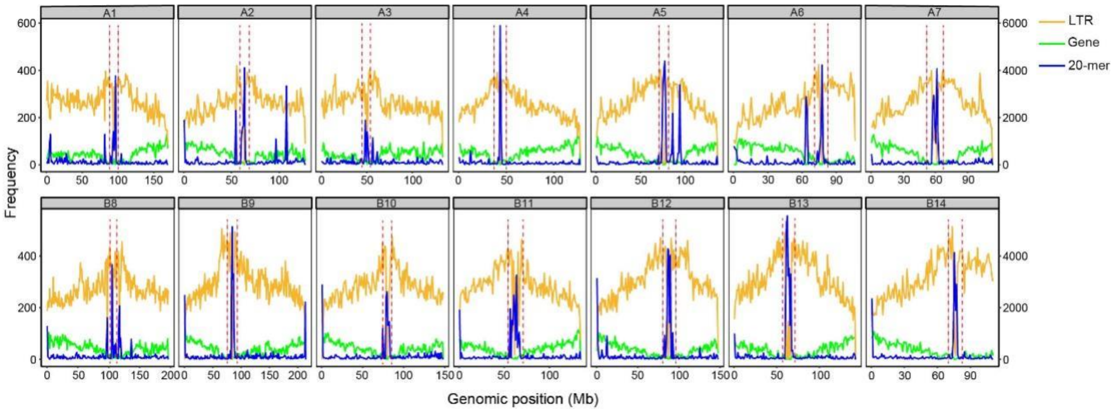


**Figure S17. Inference of centromere positions based on the distribution of three kinds of genomic features along the 14 *P. giganteum* chromosomes.** Orange curves indicate the density of long terminal repeat retrotransposons (LTR) on each chromosome of *P. giganteum* with a window size of 1 Mb. Green curves indicate the distribution protein-coding genes on each chromosome of *P. giganteum.* Blue curves indicate the distribution of the most abundant 20-kmers along each chromosome. Red dashed lines corresponds to the putative centromeric region of each *P. giganteum* chromosome.

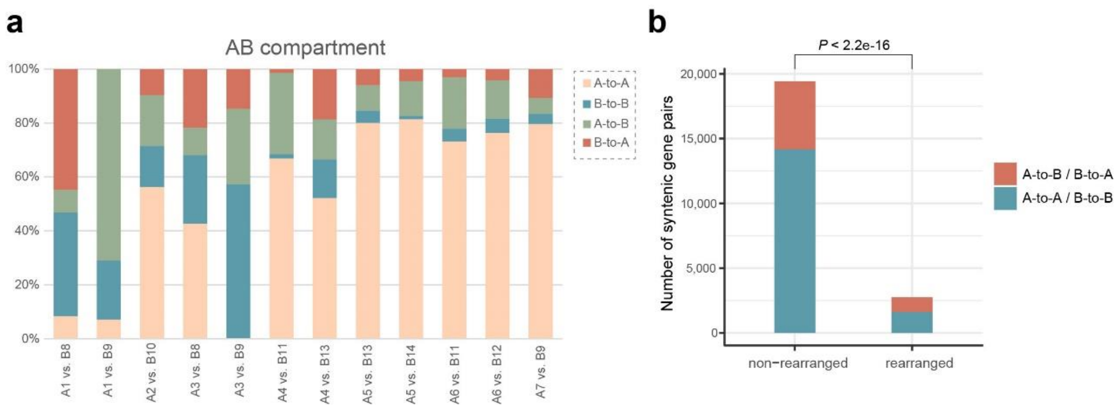


**Figure S18. Distribution of A/B compartment status conservation or switching in *P. giganteum*. (a)** Stacked bar chart showing the distribution of different types of chromatin A/B compartment status for homologous chromosome pairs in *P. giganteum*. A-to-A and B-to-B represent conserved chromatin compartment status between syntenic gene pairs, while A-to-B and B-to-A denote the switching of chromatin compartment status between syntenic gene pairs. **(b)** Comparison of A/B compartment status switching between non-rearranged and rearranged genomic regions. Statistical significance was determined using one-tailed Fisher's exact test.

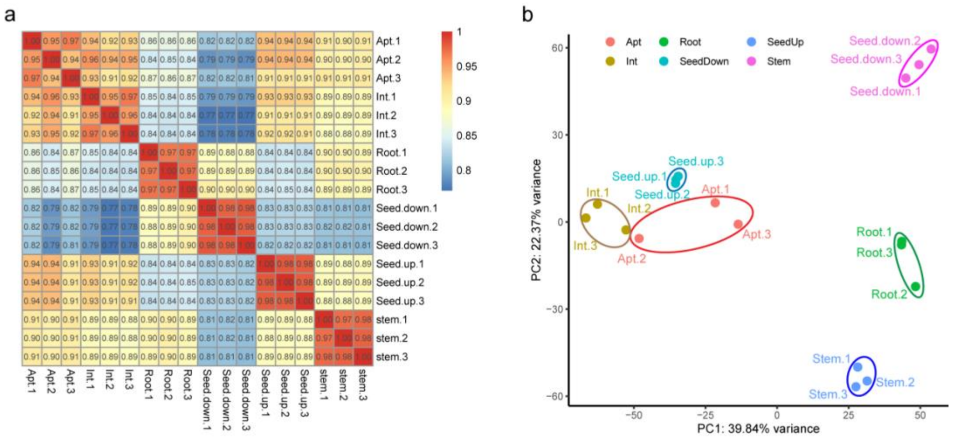


**Figure S19.** Preliminary analysis of six tissues for differential expression analysis in *P. giganteum.* **(a)** Correlation analysis for biological replicates of six tissues in *P. giganteum.* **(b)** PCA clustering of all RNA-seq samples in *P. giganteum.*

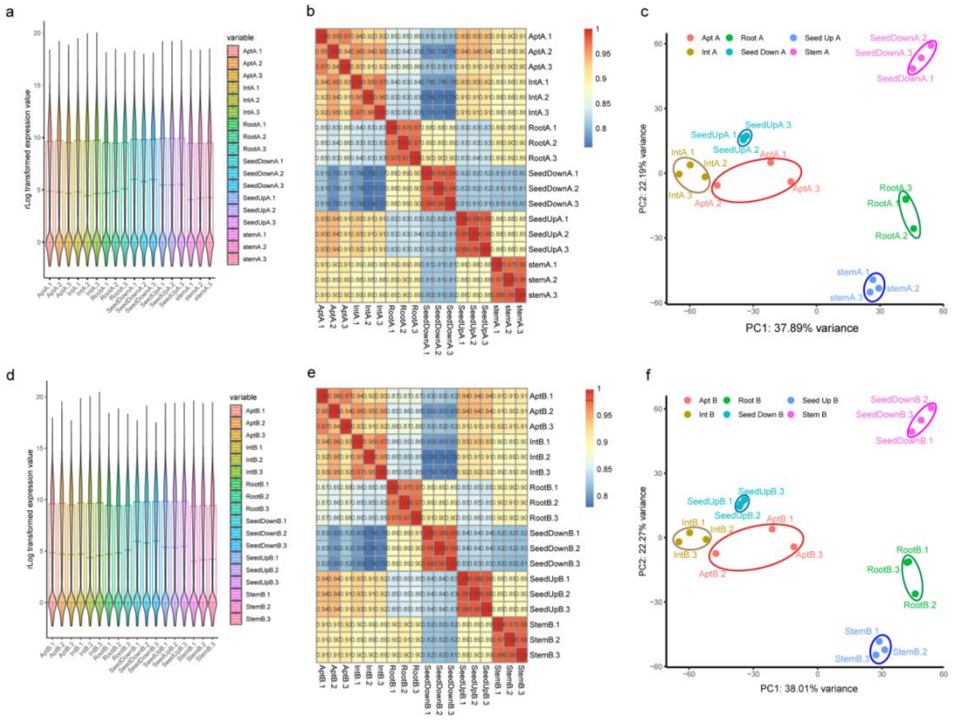


**Figure S20.** Preliminary analysis of six tissues for differential expression analysis of subgenome A and B in *P. giganteum.* Distribution of normalized expression levels in six tissues of *P. giganteum* **(a, d)**. Correlation analysis for biological replicates of six tissues in *P. giganteum* **(b, e)**. PCA clustering of all RNA-seq samples in *P. giganteum* A and B **(c, f)**.

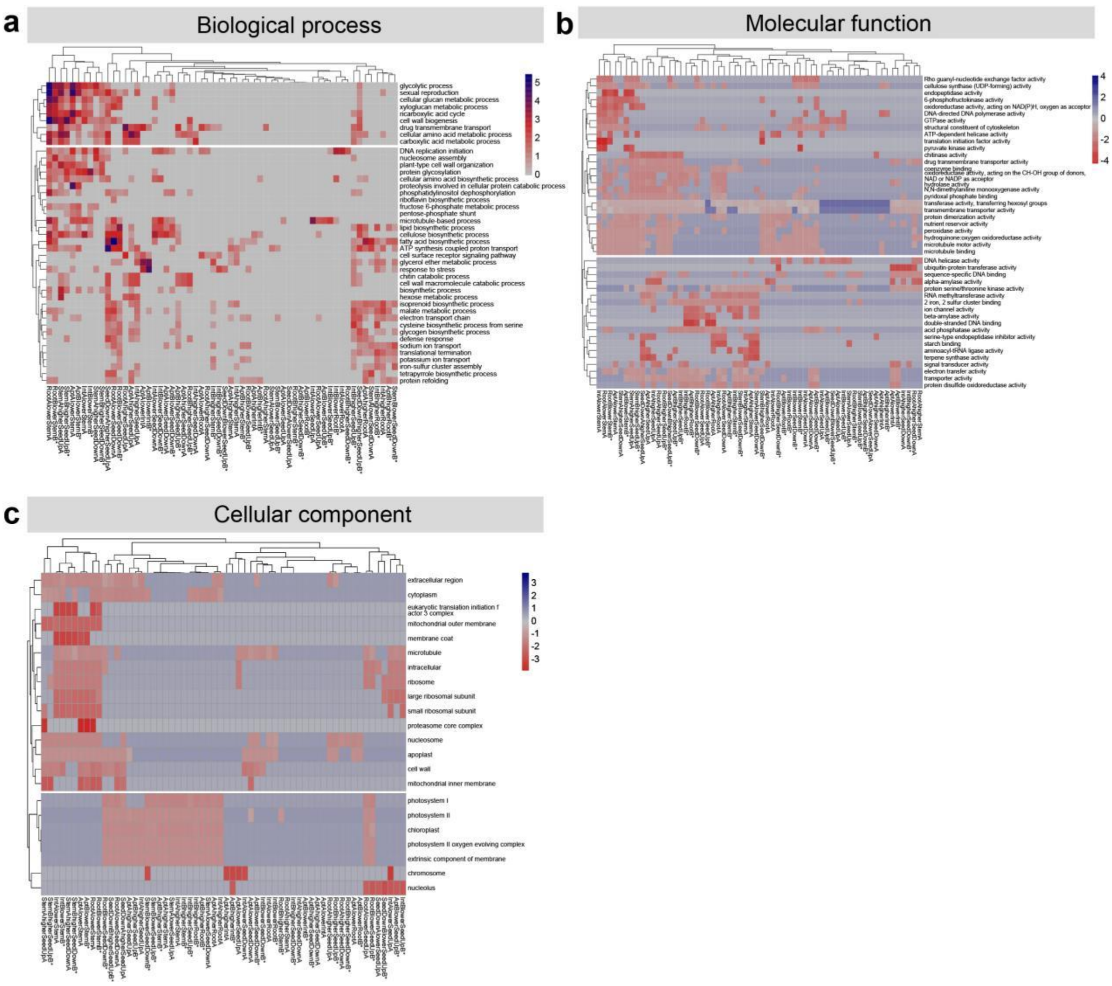


**Figure S21.** GO enrichment of differentially expressed genes (DEGs) between paired tissues in *P. giganteum*. **(a)** Enriched biological processes for DEGs between paired tissues in *P. giganteum*. **(b)** Enriched molecular functions for DEGs between paired tissues in *P. giganteum*. **(c)** Enriched cellular components for DEGs between paired tissues in *P. giganteum*.

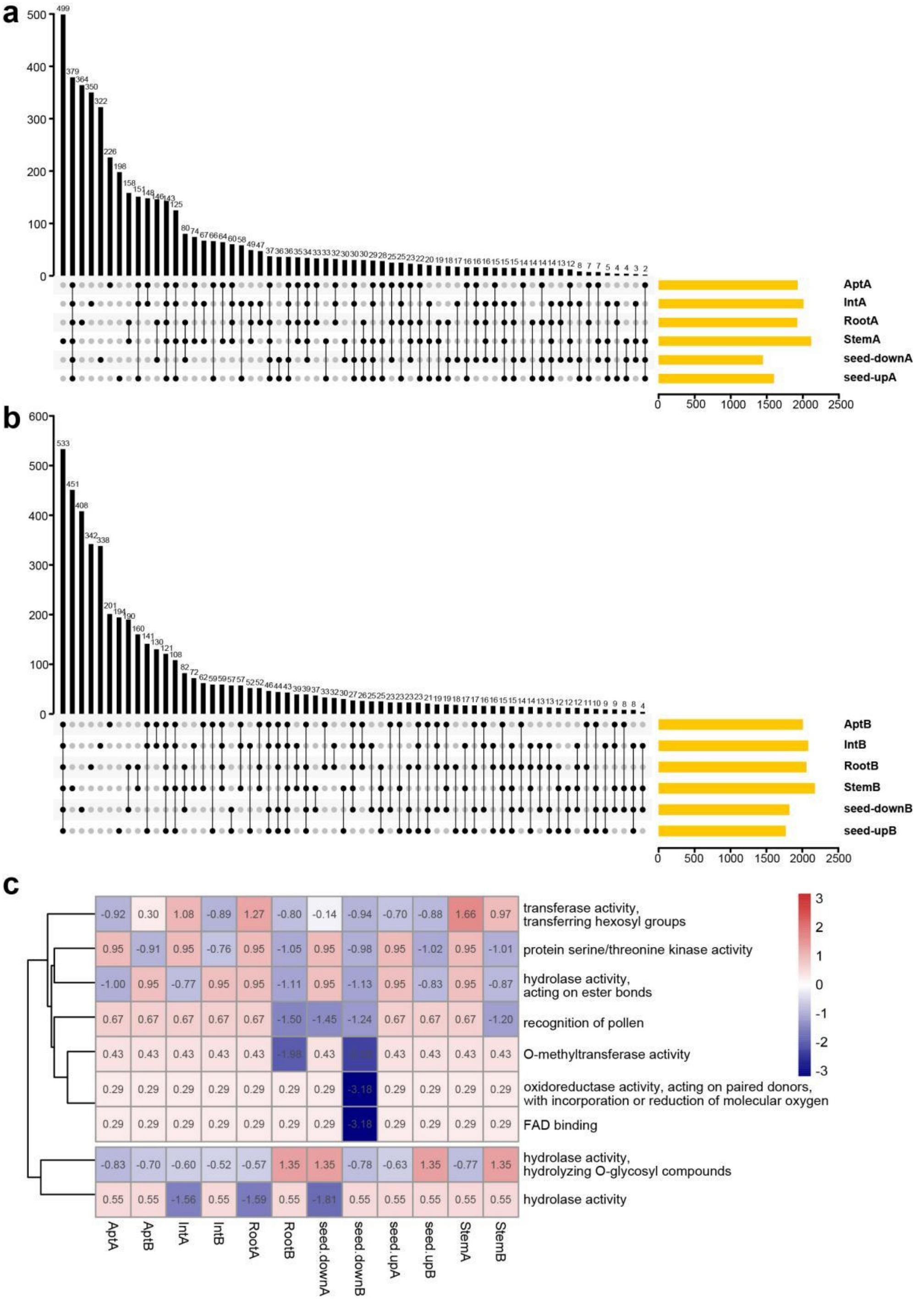


**Figure S22.** Identification of DEGs and GO enrichment analysis. **(a)** Upset plot showing DEGs among six tissues in the A subgenome of *P. giganteum*. **(b)** Upset plot showing DEGs among six tissues in the B subgenome of *P. giganteum.* **(c)** GO enrichment of DEGs of six tissues in the A and B subgenomes of *P. giganteum*.

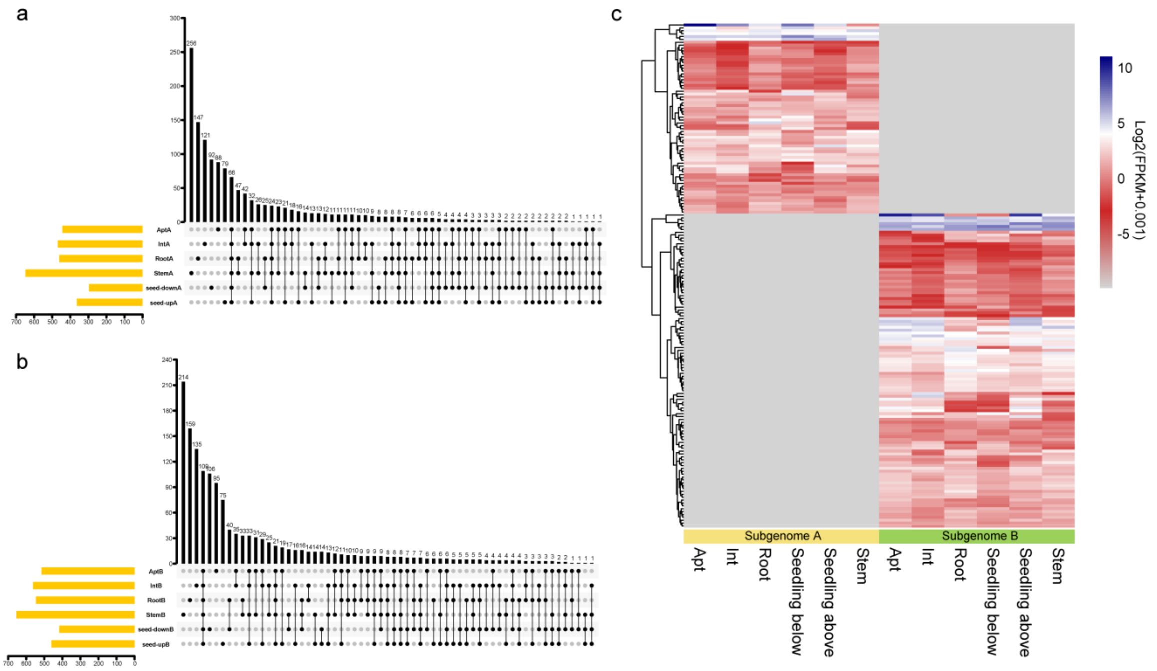


**Figure S23.** Identification of homoeologous differentially expressed genes (HDEGs) and clustering analysis. **(a)** Upset plot of HDEGs among six tissues of the A subgenome in *P. giganteum*. **(b)** Upset plot of HDEGs among six species of the B subgenome in *P. giganteum*. **(c)** Heatmap showing the expression pattern of consistent group of homoeolog-specific expressed genes (HSEGs) of six tissues in the A and B subgenomes of *P. giganteum*.

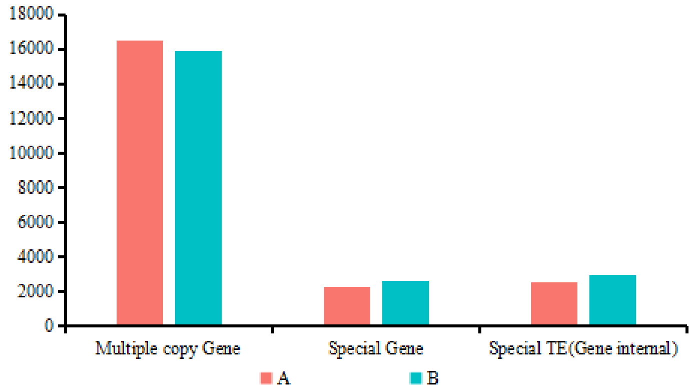


**Figure S24.** Comparison of gene level characteristics between the A and B subgenomes of *P. giganteum.*

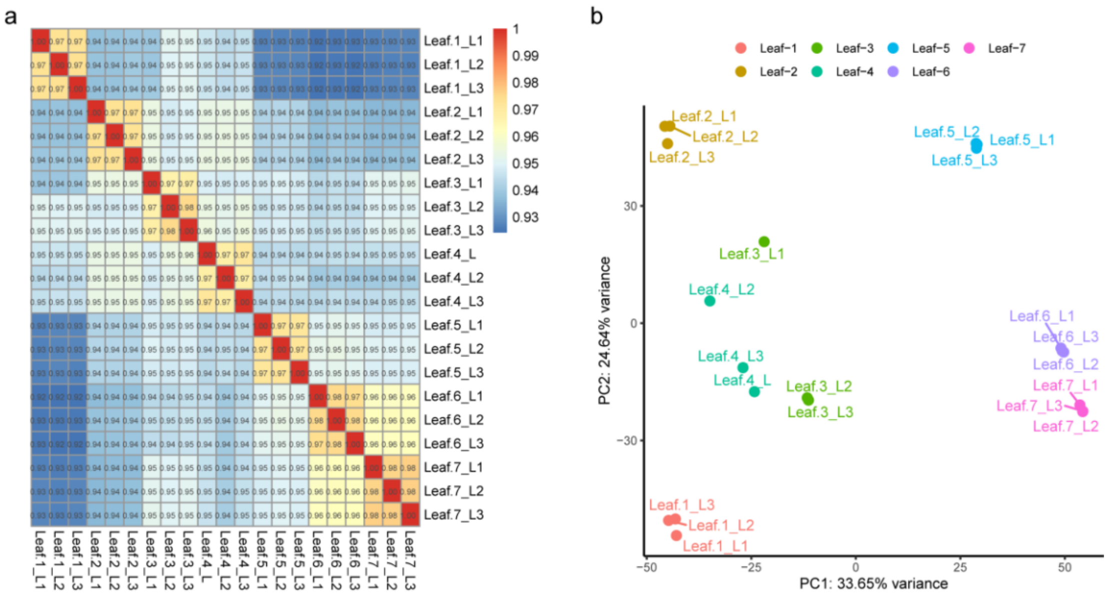


**Figure S25.** Preliminary analysis of seven stages for differential expression analysis in *P. giganteum.* **(a)** Correlation analysis for biological replicates of leaf samples at seven stages in *P. giganteum.* **(b)** PCA clustering of all leaf RNA-seq samples in *P. giganteum.*

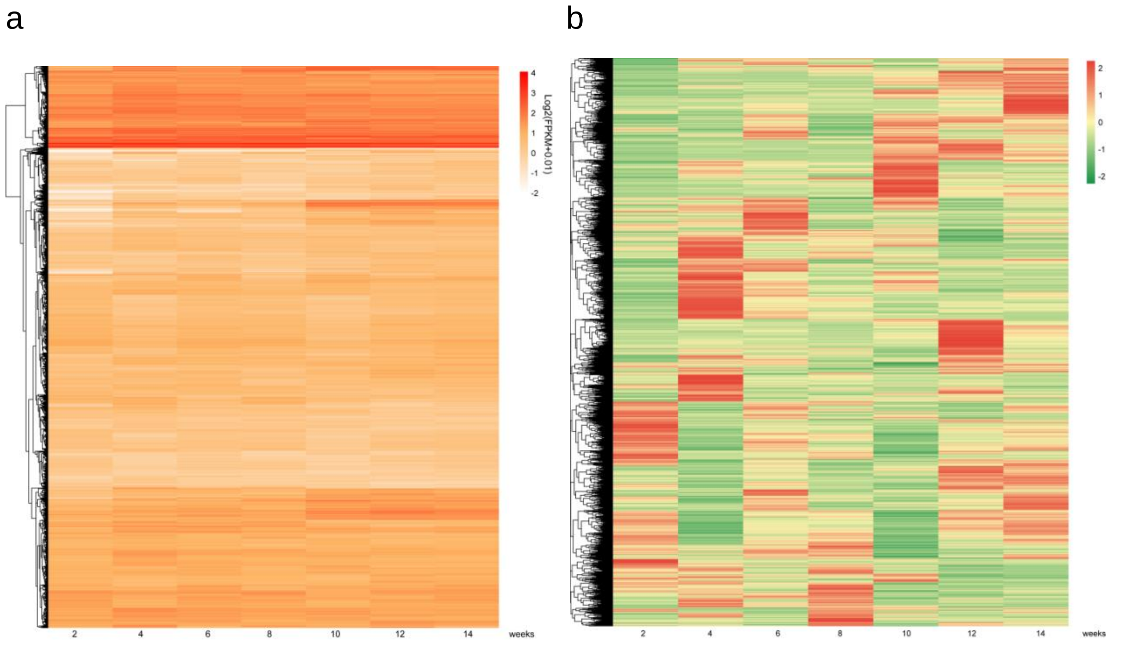


**Figure S26.** Expression patterns of differentially expressed genes in leaves at different growth stages. **(a)** The heatmap shows the log2-based FPKM+0.01 at seven growth stages. **(b)** Clustering heatmap of differentially expressed genes in leaves at different growth stages.

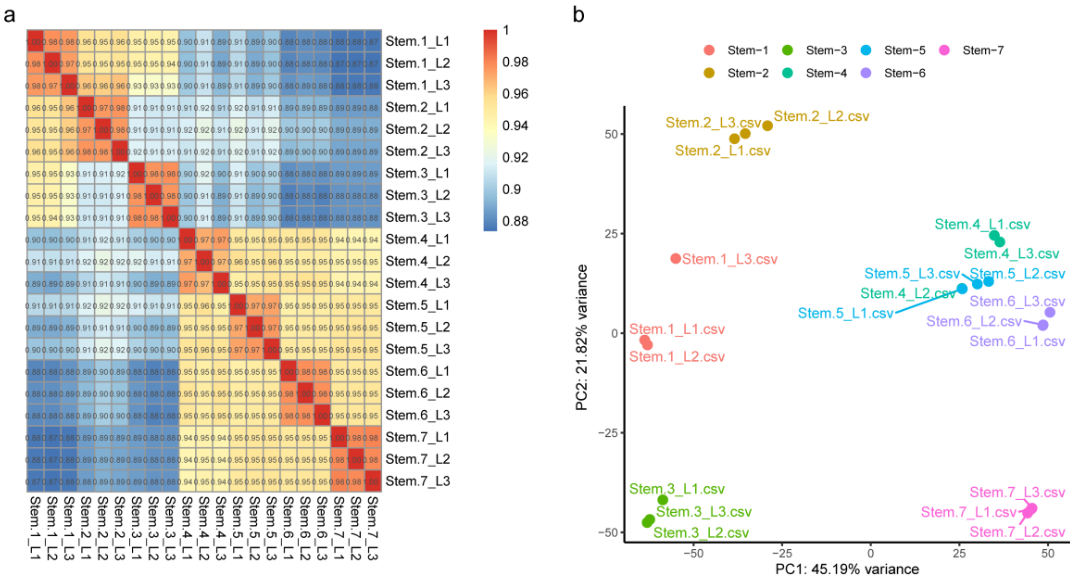


**Figure S27.** Preliminary analysis of stem RNA-seq samples at seven stages for differential expression analysis in *P. giganteum.* **(a)** Correlation analysis for biological replicates of stem samples at seven stages in *P. giganteum.* **(b)** PCA clustering of all stem RNA-seq samples in *P. giganteum.*

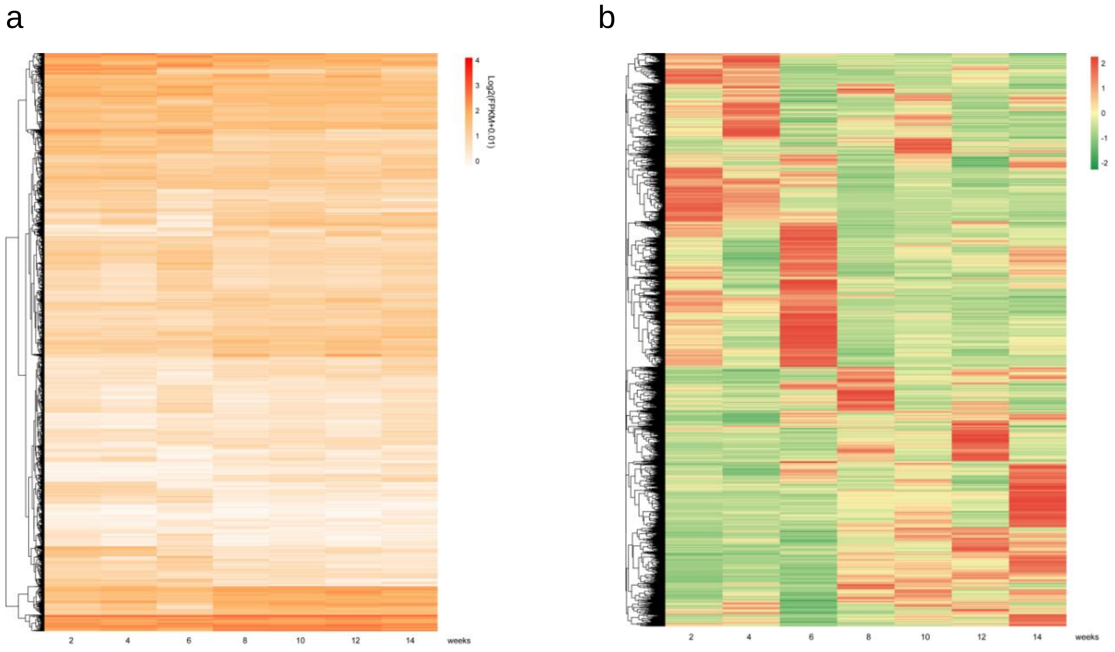


**Figure S28.** Clustering heatmap of differentially expressed genes in stems at different growth stages. Expression of differentially expressed genes in stems at different growth stages. The heatmap shows the log2-based FPKM+0.01 at seven growth stages.

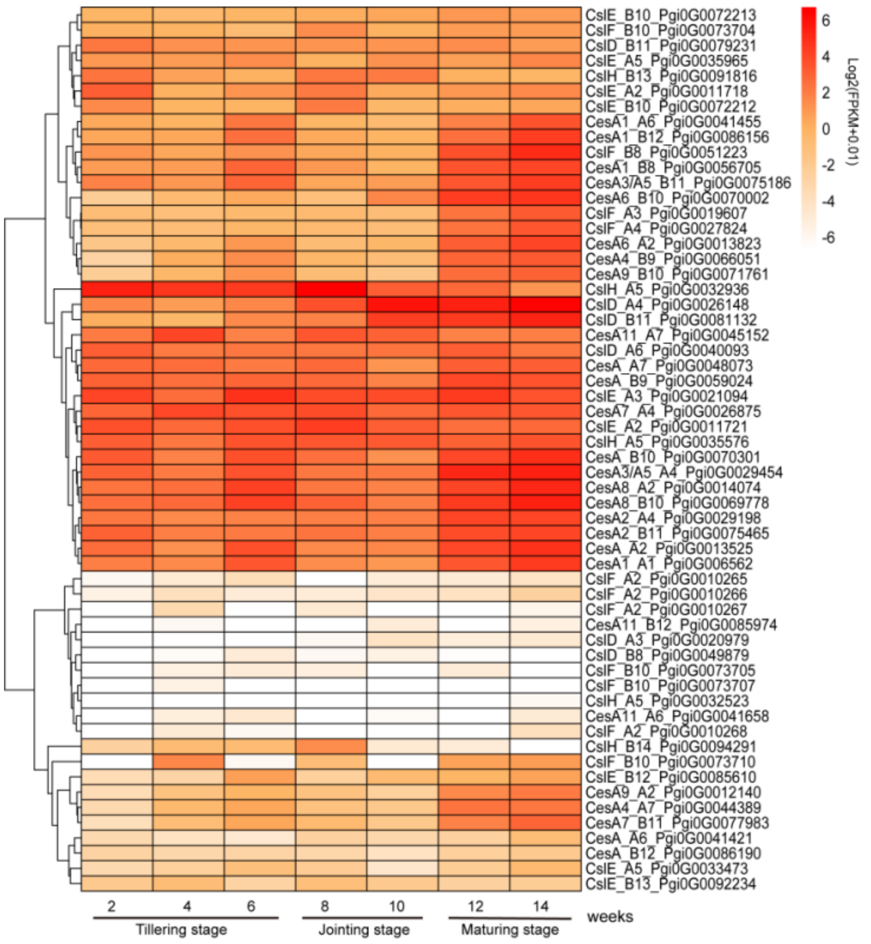


**Figure S29.** Expression of cellulose synthase-related genes in leaves at different growth stages. The heatmap shows the log2-based FPKM+0.01 at seven growth stages.

a

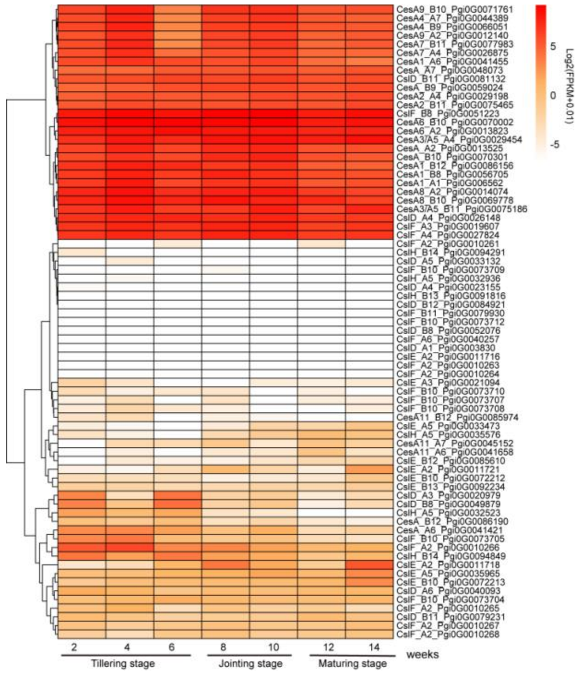


b

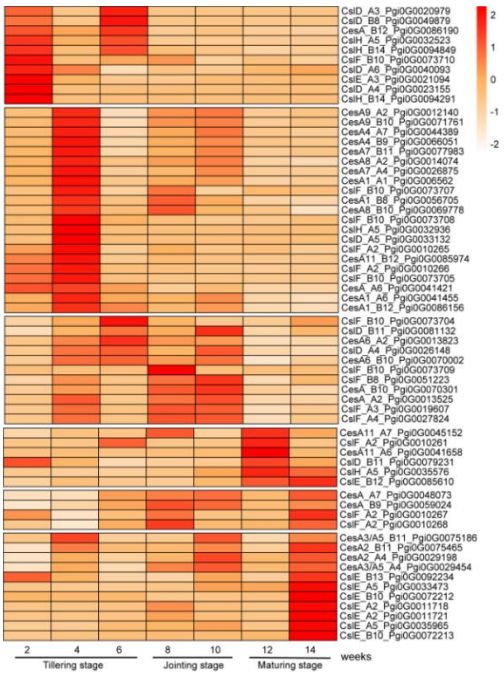


**Figure S30.** Expression of cellulose synthase-related genes in stems at different growth stages. **(a)** The heatmap shows the log2-based FPKM+0.01 at seven growth stages. **(b)** Gene expression heatmap for cellulose synthase genes that were expressed at least one time point in stems.

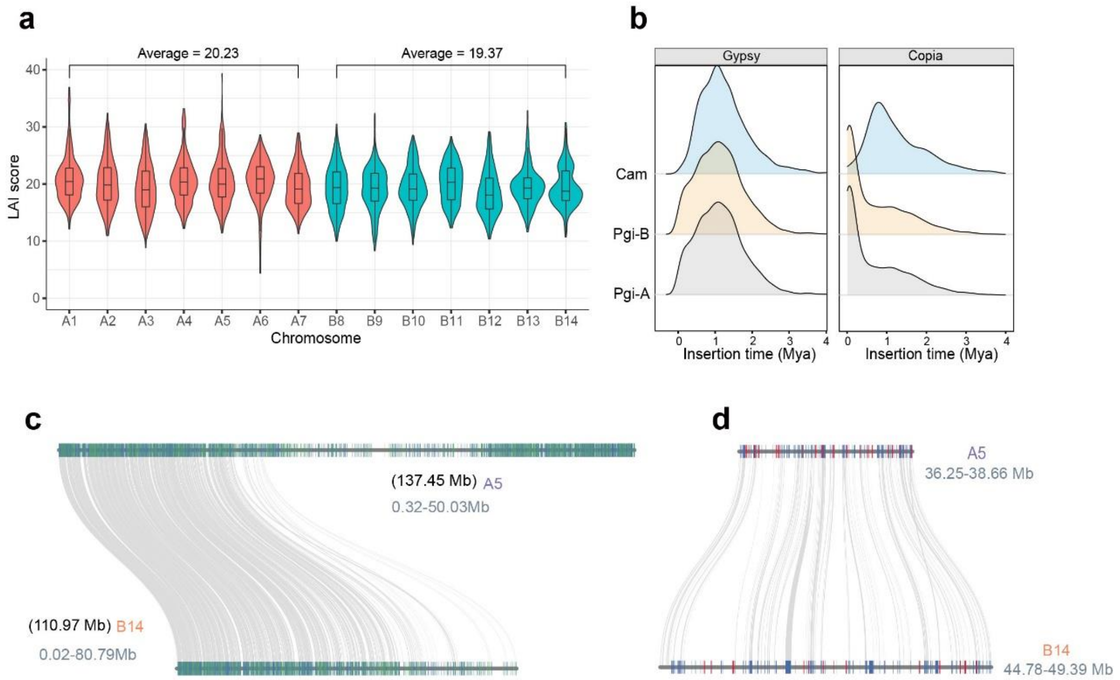


**Figure S31.** Distribution of intact LTRs in *P. giganteum* and comparison of LTRs in the syntenic region of chromosomes A5 and B14. **(a)** Boxplot showing the distribution of LTR Assembly Index (LAI) scores on each chromosome of *P. giganteum*. The gray dotted line represents the average LAI score across the whole genome. (**b)** The density plot of insertion time of *Gypsy* and *Copia* retrotransposons in C. americanus and subgenomes A and B of *P. giganteum*. The *Gypsy*-type LTR peak positions of the three species were very close, while the *Copia*-type LTR peak positions differed greatly between *C. americanus* and *P. giganteum,* thus resulting in the two peaks of LTR insertion in *P. giganteum*. **(c)** The distribution of repeat sequences in syntenic regions of A5 (0.32-50.03 Mb) and B14 (0.02-80.79 Mb). Intact LTRs are shown as green blocks and genes are indicated as blue blocks. **(d)** The distribution of repeat sequences in a microsynteny block of A5 (41.28-41.74 Mb) and B14 (66.05-66.78 Mb). Intact LTRs are shown as red blocks and other types of TEs are indicated as blue blocks.

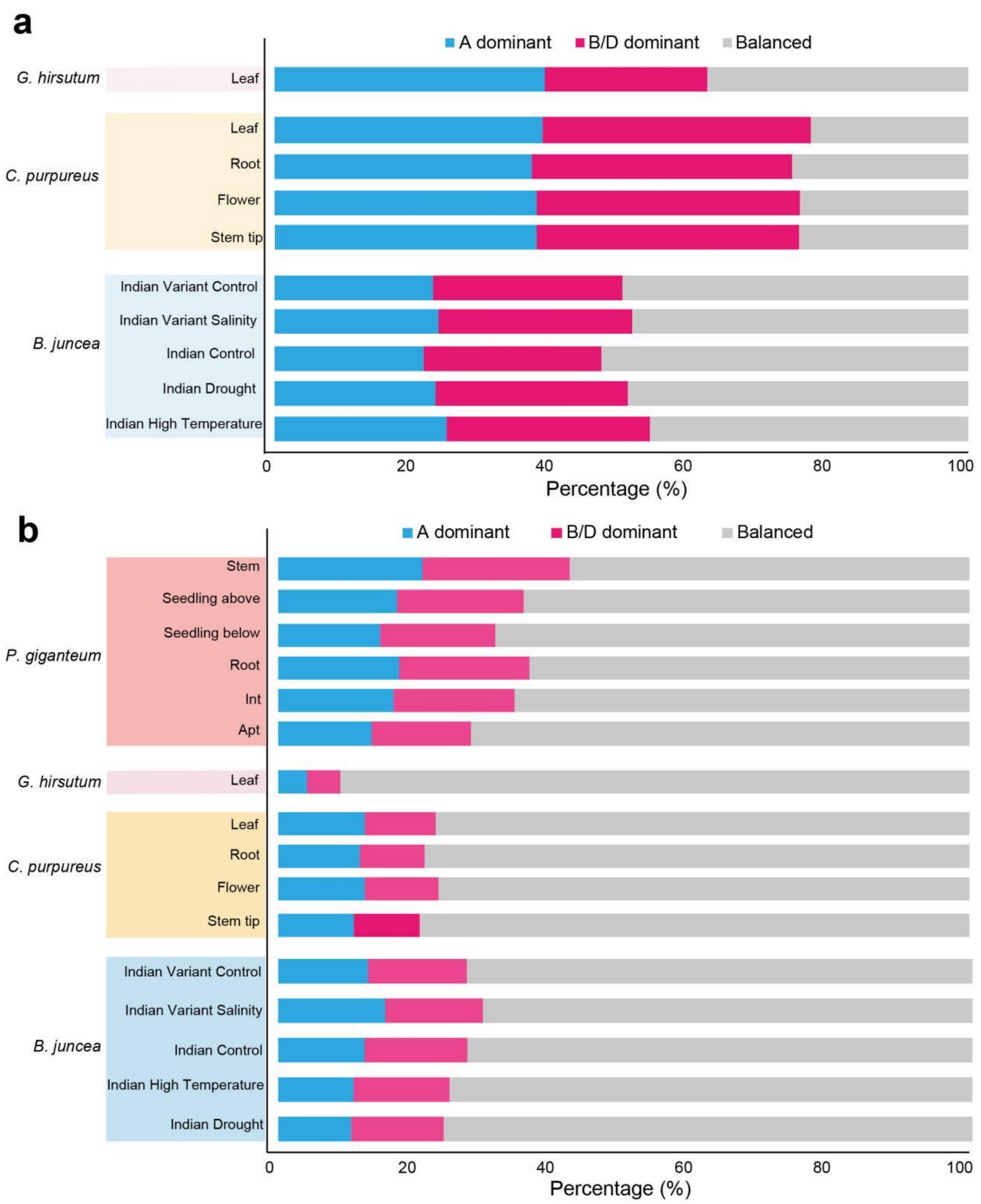


**Figure S32.** Comparison of differentially expressed genes (DEGs) and balanced genes between subgenomes across different polyploid species. **(a)** Distribution of DEGs and balanced genes between subgenomes in different studies. **(b)** Distribution of DEGs and balanced genes between subgenomes in different studies using the same method as used by us.
